# Artemisinin suppresses aerobic glycolysis in thyroid cancer cells by downregulating HIF-1a, which is increased by the XIST/miR-93/HIF-1a pathway

**DOI:** 10.1371/journal.pone.0284242

**Published:** 2023-04-10

**Authors:** Fei Yang, Jie Zhang, Zhijun Zhao, Yan Liu, Zhen Zhao, Kai Fu, Baokun Li, Jing Jin

**Affiliations:** 1 Department of Otolaryngology Head and Neck Surgery, The Fourth Hospital of Hebei Medical University, Shijiazhuang, China; 2 The Second General Surgery, The Fourth Hospital of Hebei Medical University, Shijiazhuang, China; 3 Cancer Institute, Fourth Hospital of Hebei Medical University, Shijiazhuang, China; King Faisal Specialist Hospital and Research Center, SAUDI ARABIA

## Abstract

The incidence of thyroid cancer (TC) continues to increase worldwide. Aerobic glycolysis, the prominent feature of glucose metabolism in cancer progression, is associated with TC. We first demonstrated that HIF-1a is highly expressed in TC tissues and is positively correlated with the level of XIST in the serum of patients with TC. Then, we proved that XIST regulates the expression of HIF-1a through the XIST/miR-93/HIF-1a pathway, thereby regulating the level of glycolysis in TC cells. Knockdown of XIST inhibits glycolysis, proliferation, the cell cycle and metastasis of TC cells. Finally, we verified that artemisinin could target the degradation of HIF-1a and inhibit glycolysis in TC cells. Collectively, XIST levels in serum may be used as a tumor marker for TC promoted by HIF-1a, which could be treated using artemisinin.

## Introduction

The prevalence of thyroid cancer (TC), which is a malignant endocrine tumor, continues to increase worldwide [1]. According to Global Cancer Statistics 2020, there were more than 586,000 new cases of TC in 2020, ranking 11th in cancer incidence [2]. Papillary thyroid carcinoma (PTC) is the most prevalent type of thyroid carcinoma, accounting for 80% of all thyroid carcinomas [3,4]. Although the precise causes of TC remain unclear, a few risk factors, such as exposure to radiation, sex (women), and a diet low in iodine (follicular thyroid cancer), are known to increase the risk of TC [5]. In addition, the gene mutation is closely related to the occurrence and development of thyroid carcinoma. Studies have shown that there is a high correlation between TERT promoter mutation and invasive thyroid carcinoma [6], between ALK gene mutation and anaplastic thyroid carcinoma [7], and between isocitrate dehydrogenase 1 (IDH1) and thyroid carcinoma [8]. However, unlike other tumors, GNAQ, MMP8, AKT3, EGFR, and PIK3R1 mutations are not common in thyroid carcinoma [9]. Other studies have shown that aerobic glycolysis is closely related to the occurrence and development of TC.

Aerobic glycolysis, the prominent feature of glucose metabolism in cancer progression, is associated with the progression of multiple cancers and could support sustained malignant cancer cells [10]. Aerobic glycolysis has also been shown to be associated with TC [11]. However, there are few related studies, and the regulatory mechanism of aerobic glycolysis in TC is still unclear. HIF-1a is a determinant of aerobic glycolysis in tumor cells [12] and is crucial in glucose metabolism, proliferation, and invasion in cancer [13]. Studies have shown that the expression of HIF-1a is regulated by the lncRNA XIST [14] and that the lncRNA XIST is also associated with TC [15]. XIST is a noncoding RNA expressed only in the inactive X chromosome and is the key molecular element initiated by the X-inactivation center (XIC). It is located in Xq13.3 [16]. Long-stranded noncoding RNAs (lncRNAs) participate in the regulation of key cellular genes in cell differentiation, proliferation, the cell cycle, apoptosis, migration, and invasion [17] mainly by regulating the expression of thyroid oncogenes. In this study, we will verify the mechanism that XIST regulates glycolysis of thyroid cancer cells through HIF-1a, and explore XIST as a biomarker of thyroid cancer progression and corresponding intervention measures.

## Materials and methods

### Patients and samples

All enrolled patients underwent surgical treatment at The Fourth Hospital of Hebei Medical University (also known as The Tumor Hospital of Hebei Province, which is a large and comprehensive level three, grade A hospital) from January 1, 2020, to December 31, 2020. Serum specimens were obtained from patients prior to surgery. The inclusion criteria were that the patient did not previously undergo radiation therapy and chemotherapy. A total of 40 patients who were admitted met the inclusion criteria. Overall, 14 patients were male, and 26 patients were female. The median age was 48 years of age (range 35–74 years).

### Cell culture

The human TC cell line TPC-1 and normal thyroid cell line Nthy-ori 3–1 were purchased from Shanghai Zhongqiaoxinzhou Biotech, China. The cells were cultured in RPMI-1640 medium (HyClone, Logan, Utah, USA) with 10% heat-inactivated fetal bovine serum (FBS, PAN, Adenbach, Germany) at 37°C in a 5% CO_2_ humidified incubator. TPC-1 cells were plated approximately 16 hours before transfection. Lentivirus for knockdown of XIST was ordered from Genehem (Shanghai, China), and the plasmid sequence of sh-XIST was as follows: 5’-TCTCTGTCATTGCTTCTGTAGTCACAGTC -3’. According to the results of preliminary experiments, the concentration of artemisinin in this study was 10 μmol/l. Cell viability was detected using MTS.

### Immunohistochemical assay

Paraffin sections (4-mm thick) were deparaffinized and rehydrated, followed by treatment with 0.02 M EDTA buffer (pH 9.0, Gene Tech, USA). Then, the sections were immersed in 3% H_2_O_2_ and blocked with 5% normal goat serum in the proper sequence, followed by incubation with monoclonal anti-HIF-1a antibody (1:500; Abcam, USA) overnight at 4°C. The antibody was diluted in PBS buffer containing 5% normal goat serum. The negative control for each slide was incubated with 5% normal goat serum without the anti-HIF-1a antibody. The sections were then incubated with HRP-conjugated anti-rabbit IgG (ZSGB-BIO, China) for 45 min at 37°C and revealed with diaminobenzidine tetrahydrochloride. The stained slides were scored by three pathologists who were unaware of the clinical diagnosis. The indices of HIF-1a labeling were implemented so that samples were scored according to the percentage and intensity scores of positively stained tumor cells.

### Quantitative real-time PCR

Total RNA was extracted from tissue samples according to the instructions in the manual form iRNAVanaTM PARISTM (Ambion, TX, USA). Briefly, 10 ng of total RNA was used as the template for 15 μl reverse transcription reactions. For each RNA, reactions were performed in triplicate using the 7500 RT‒PCR system (Applied Biosystems), and RNU66 (Applied Biosystems, cat. no. 4373382) was used as the normalization control. The sequence of the gene was as follows:

**Table:** 

Gene	Forward sequences	Reverse sequences
XIST	5´-TGGCTCTTCTTTCACGCTTT-3´	5´-TGGTGTCGTGGAGTCG-3´
U6	5´-CTCGCTTCGGCAGCACA-3´	5´-AACGCTTCACGAATTTGCGT-3´

### Western blot analysis

Cells were lysed using precooled RIPA lysis buffer on ice for 45 minutes, and then the lysate was centrifuged at 12,000 rpm for 10 minutes at 4°C. The concentration of the extracted protein was determined using the BCA protein concentration determination kit, 50 μg of protein was loaded on the gel, separated by SDS‒PAGE gel, and transferred to the PVDF membrane. The membrane was blocked with 5% skimmed milk powder at room temperature for 1 hour and incubated with antibodies (anti-GAPDH, 1:10000; anti-HIF-1α, 1:500) at 4°C overnight. A concentration of 0.1% of Tween-containing TBST was washed 3 times and incubated with horseradish peroxidase-linked secondary antibody (1:2,000) for 1.5 hours at room temperature. The membrane was washed 3 times with TBST containing 0.1% Tween, and the immunoreactive bands were detected using a scanner (Odyssey Infrared Imaging System (LI–COR, USA)).

### Luciferase reporter assay

The predicted 3’UTR sequences of XIST and HIF-1, which bind to miR-93-5p, were cloned from the genomic DNA of TC cells. Then, the sequence was inserted into the pmir-GLO control luciferase reporter vector. Luciferase reporter assays were conducted using the Dual-Glo luciferase assay system (Shanghai Genechem, China).

### Seahorse assays

A Seahorse Extracellular Flux (XF96e) Analyzer and the Agilent Seahorse XF Glycolytic Rate Assay Kit were used to measure the extracellular acidification rate (ECAR), reflecting the glycolytic level of live esophageal cancer cells. The specific experimental procedures were performed according to the manufacturer’s instructions.

### Flow cytometry assays (FCM)

Cells were harvested and washed twice with FBS and then fixed in a 70% ethanol solution for 24 h at 4°C. Then, the fixed cells were washed once with PBS and resuspended as a single-cell suspension. The suspension was incubated with 500 μl of PI (50 μg/ml) for 30 min and tested by FCM according to standard procedures.

### Molecular docking

We downloaded the protein molecule using the PDB database, and artemisinin was downloaded through the TCMSP platform. AutoDock4.2 was used for Molecular Docking. PyMOL and Ligplot were used for visualization. See our previous article for the specific operation procedure [18].

### Statistical analysis

All statistical analyses were performed using SPSS 21.0 software (SPSS Inc., USA). Quantitative results are shown as the mean ± SD. The Student-Newman‒Keuls test was used for statistical analyses between the groups. P<0.05 was considered statistically significant.

## Results

### Expression of HIF-1a in TC tissues was significantly higher than that in paracarcinoma tissues and was positively correlated with XIST

**Fig 1:**
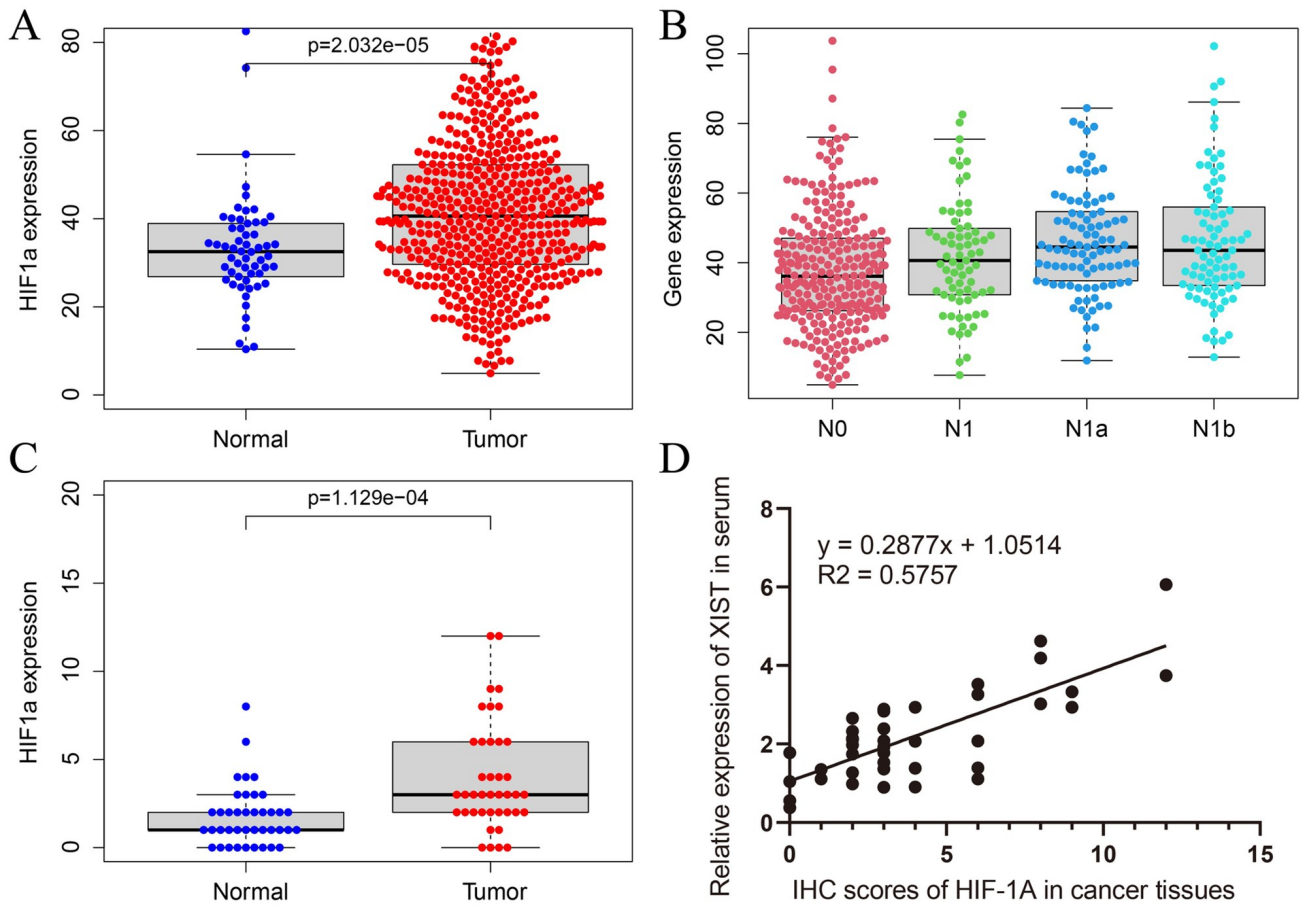
Expression levels of HIF-1a in TC tissues. (A) The expression of HIF-1a in TC tissues and adjacent tissues from TCGA database. (B) The expression of HIF-1a in lymph node metastasis of TC from TCGA database. (C) HIF-1a expression in TC and adjacent tissues by IHC. (D) Correlation between the expression levels of HIF-1a in TC tissues and the expression levels of XIST in serum.

HIF-1a RNA-seq gene expression data in cancer tissues and adjacent tissues of 570 TC patients were collected in the The Cancer Genome Atlas (TCGA) database (58 normal tissues and 512 cancer tissues, S1 Table, https://github.com/416057233/Artemisinin_thyroid-cancer_2023.git). The results showed that HIF-1a was highly expressed in cancer tissues (Fig 1A). High expression of HIF-1a was associated with lymph node metastasis (Fig 1B). We examined HIF-1a protein expression levels in tissues by IHC and XIST expression levels in serum by qRT‒PCR. The results showed that compared with the expression levels in adjacent tissues, HIF-1a protein levels were significantly upregulated in TC tissues (Fig 1C, S2 Table, https://github.com/416057233/Artemisinin_thyroid-cancer_2023.git). The relative XIST expression level in serum was detected using U6 as a standard and was positively correlated with HIF-1a expression levels in cancer tissues (*P*<0.001) (Fig 1D).

### XIST regulated glycolysis, proliferation, the cell cycle and metastasis of TC cells

**Fig 2:**
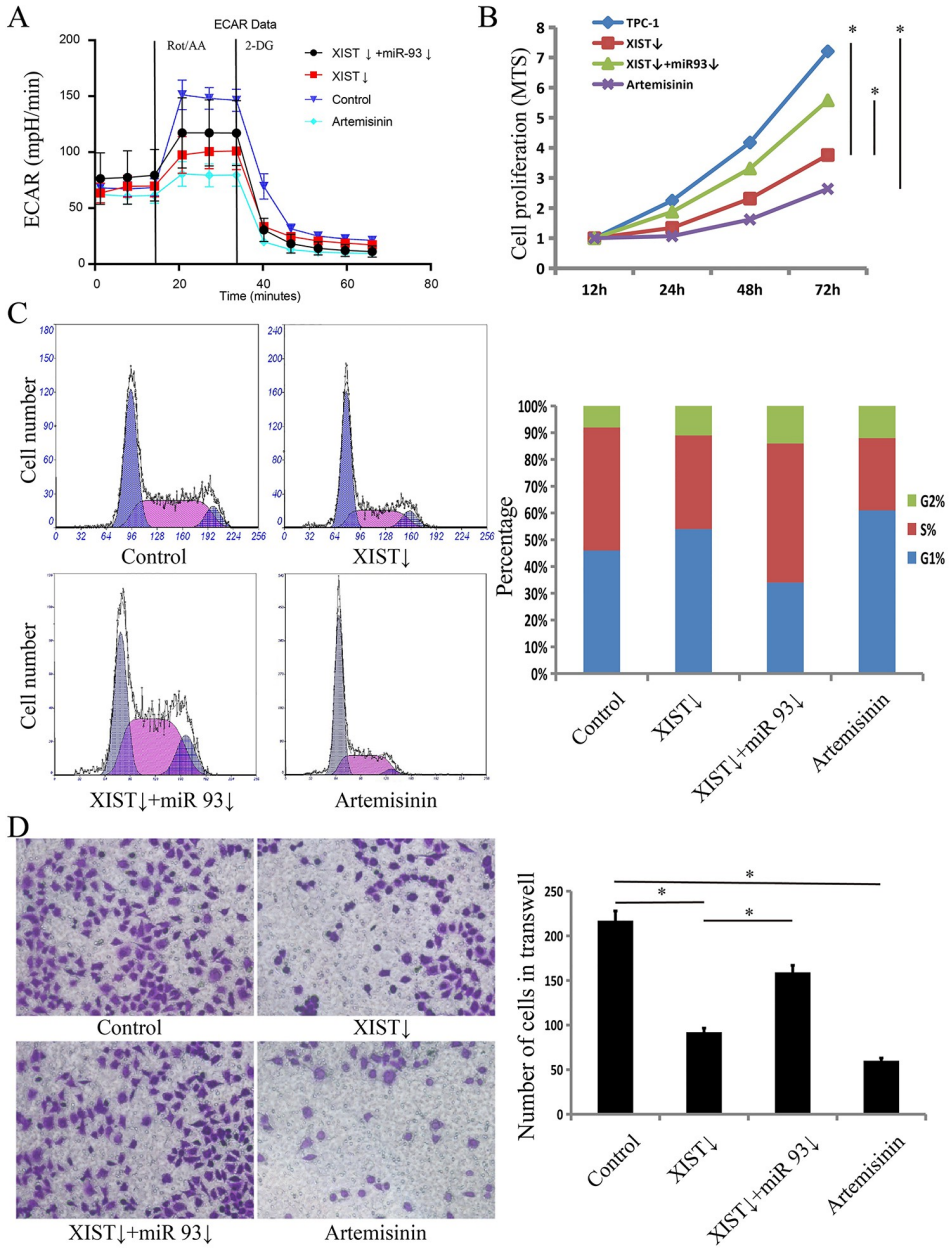
The role of XIST, miR-93 and artemisinin in TC cells. (A) The effect of XIST, miR-93 and artemisinin on the aerobic glycolysis of TC cells by ECAR. (B) The effect of XIST, miR-93 and artemisinin on the viability of TC cells by MTS. (C) The effect of XIST, miR-93 and artemisinin on the cell cycle of TC cells by flow cytometry. (D) The effect of XIST, miR-93 and artemisinin on the metastasis of TC cells by Transwell assays.

Because the role of HIF-1a in cancer is to promote aerobic glycolysis, we tested the effects of XIST on the level of aerobic glycolysis in TC cells by ECAR. The results showed that knockdown of XIST significantly inhibited aerobic glycolysis levels (Fig 2A). Then, we examined the proliferation, cell cycle and metastasis of TC cells. According to the MTS experimental results, knockdown of XIST reduced cell proliferation by 48% (Fig 2B). According to the FCM experimental results, knockdown of XIST inhibited the cell cycle, and G1 phase cells increased by 17% (Fig 2C). According to the Transwell experimental results, knockdown of XIST reduced metastasis by 51% (Fig 2D). Knockdown of XIST could inhibit aerobic glycolysis, proliferation, the cell cycle and metastasis of TC cells.

### XIST regulates the expression of HIF-1a through the XIST/miR-93/HIF-1a pathway

**Fig 3:**
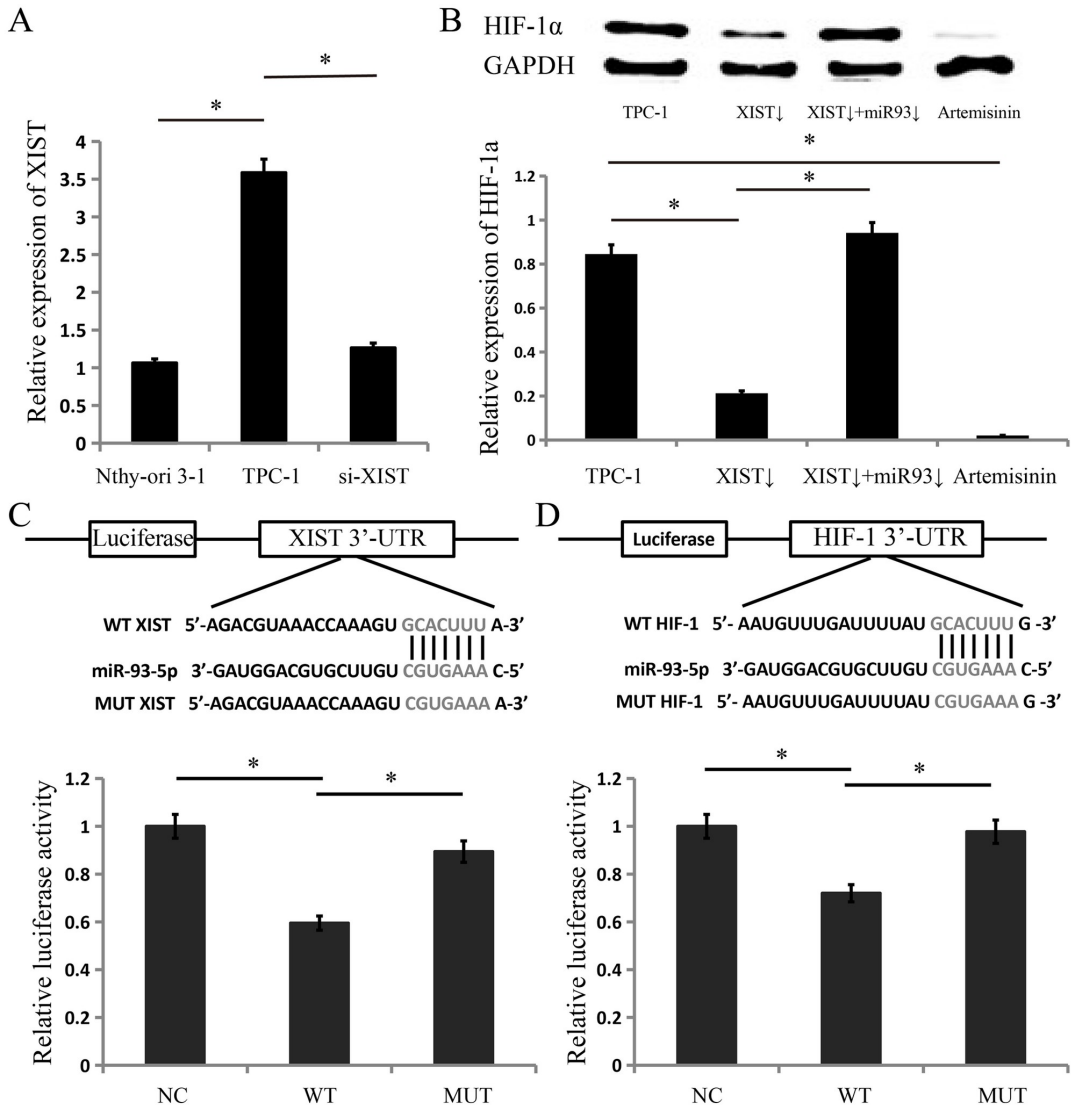
XIST inhibits the expression of HIF-1a by binding to miR-93-5p. (A) Relative XIST levels of cell lines were measured according to the standard value of the normal thyroid cell line Nthy-ori 3–1, which was 1. (B) Knockdown of XIST inhibited the expression of HIF-1a, and knockdown of miR-93-5p increased the expression of HIF-1a. Artemisinin inhibited the expression of HIF-1a in B-CPAP cells. (C) The direct binding of the XIST 3’-UTR and miR-93-5p was detected using luciferase reporter assays. (D) The direct binding of HIF-1 mRNA 3’-UTR and miR-93-5p was detected by luciferase reporter assays.

The expression level of XIST was correlated with the expression level of HIF-1a, and we further verified the regulatory relationship between XIST and HIF-1a. We first detected the expression level of XIST in TPC-1 human TC cells and Nthy-ori 3–1 normal thyroid cells, and the results showed that XIST was significantly highly expressed in TPC-1 cells. We downregulated XIST (Fig 3A) in the TC cell line TPC-1 and found that the expression level of HIF-1a was reduced (Fig 3B). We explored the mechanism by which XIST increases the expression level of HIF-1a and identified HIF-1a as one of the targets of miR-93-5p through searches of the “TargetScan” and “miRanda” databases. XIST may bind to miR-93-5p as a ceRNA. We performed luciferase reporter assays to determine whether XIST combined with miR-93-5p directly and whether miR-93-5p inhibits the activity of HIF-1 mRNA by binding to the target sites. The results showed that compared with the control group, the miR-93-5p mimic suppressed the luciferase activity of psiCHECK2 containing XIST (Fig 3C) and HIF-1a (Fig 3D). The results indicate that XIST regulates HIF-1a by combining with miR-93-5p as a sponge.

Then, we validated the XIST/miR-93/HIF-1a pathway. We downregulated miR-93 in the cells with knockdown of XIST, and the western blot results showed that downregulation of miR-93 in the cells with knockdown of XIST could restore the expression of HIF-1a (Fig 3B). According to the ECAR experimental results, downregulated miR-93 in the cells with knockdown of XIST could increase aerobic glycolysis levels (Fig 2A). According to the MTS experimental results, downregulation of miR-93 increased cell proliferation by 25% (Fig 2B). According to the FCM experimental results, downregulation of miR-93 could promote the cell cycle, and G1 phase cells decreased by 39% (Fig 2C). According to the Transwell experimental results, downregulation of miR-93 increased metastasis by 88% (Fig 2D). Knockdown of XIST could inhibit aerobic glycolysis, proliferation, the cell cycle and metastasis of TC cells. Downregulation of miR-93 in cells with knockdown of XIST restored aerobic glycolysis, proliferation, the cell cycle and metastasis, proving that XIST works through the XIST/miR-93/HIF-1a pathway.

### Artemisinin promoted the degradation of HIF-1a

**Fig 4:**
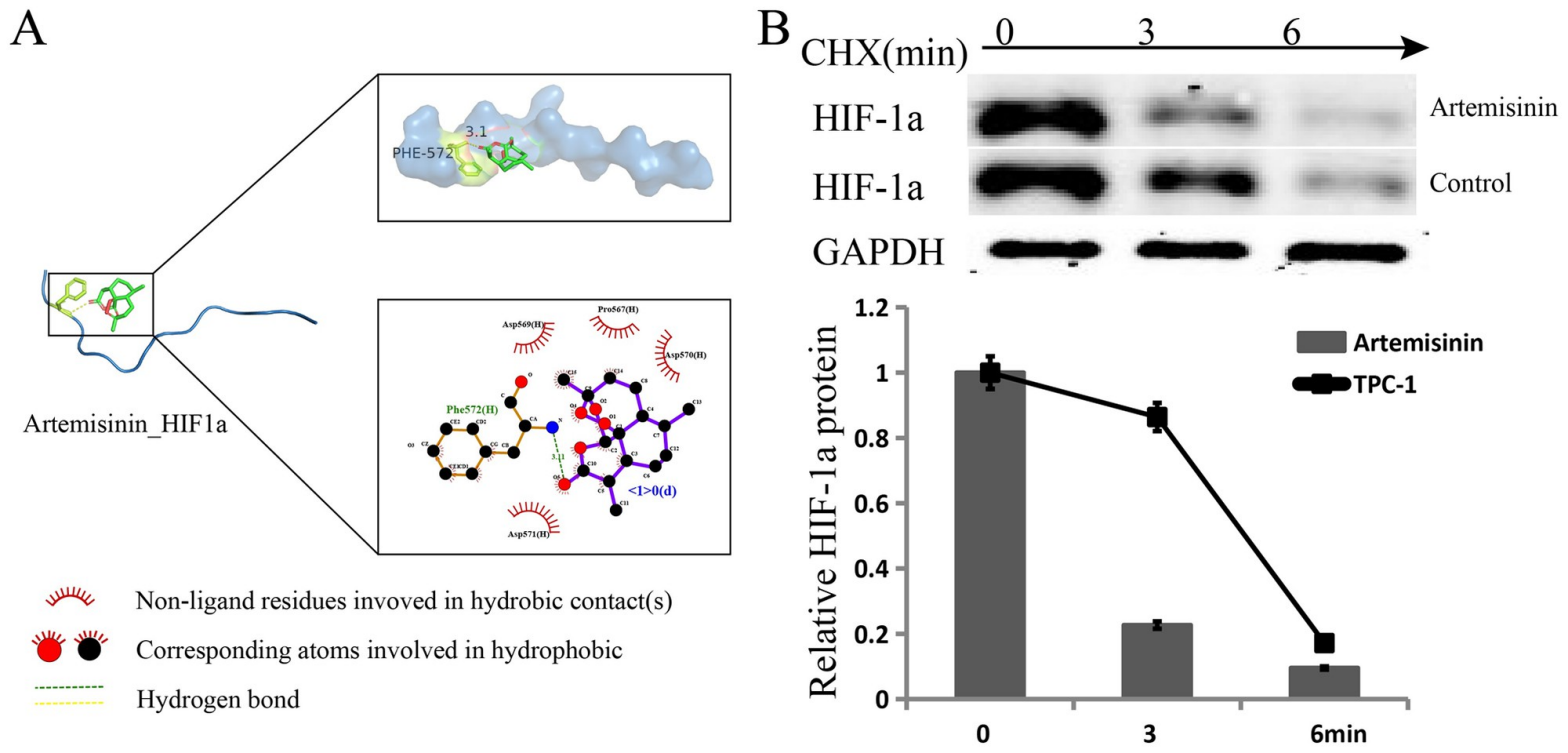
Artemisinin targets and degrades HIF-1a. (A) The binding site of artemisinin and HIF-1a. (B) The half-life of HIF-1a protein was assessed in TC cells. Cells were treated with artemisinin.

A search of TCMSP and PharmMapper revealed that artemisinin was found to target HIF-1a, and we simulated the interaction between artemisinin and HIF-1a through molecular docking. The link between artemisinin and HIF-1a is shown in Fig 4A, and the binding energy was -4.5 kcal/mol, indicating that it could bind spontaneously. We tested the effect of artemisinin on the expression of HIF-1a, and the results showed that artemisinin inhibited the expression of HIF-1a (Fig 3B). To determine how the HIF-1a protein is inhibited by artemisinin, we used cycloheximide (CHX) and analyzed the stability of HIF-1a. The results showed that the HIF-1a protein degradation rate was promoted by artemisinin (Fig 4B). We further tested the role of artemisinin in TC cells and found that artemisinin could inhibit aerobic glycolysis, proliferation, the cell cycle and metastasis (Fig 2A–2D).

## Discussion

Cancer cells have faster growth and metabolism. Compared with normal cells, cancer cells have a unique mechanism of glycometabolism. As early as 1956, Warburg discovered that tumor tissue undergoes glycolysis even under conditions of sufficient oxygen, which is called the "Warburg" effect, or aerobic glycolysis [19]. Aerobic glycolysis can produce a large number of metabolites while rapidly producing a large amount of ATP. These energy sources and metabolites can promote cancer progression [20]. HIF-1a is a determinant of aerobic glycolysis in tumor cells, and hypoxia increases the expression of HIF-1a. The overexpression of HIF-1a caused by hypoxia could promote glycolysis when the oxygen partial pressure is lower than 10 mm [21,22]. The expression of HIF-1a is also affected by other factors, such as insulin levels, lactate levels, pyruvate levels and genetic changes (such as oncogene activation or tumor suppressor gene inactivation) [23,24]. Studies have shown that the expression of HIF-1a is regulated by lncRNA XIST [14]. XIST was shown to be abnormally expressed in TC. Liu et al. confirmed that XIST serves as a ceRNA for miR-34a, competing with MET for miR-34a binding, and promotes TC cell proliferation and tumor growth [15]. Du et al. found that the lncRNA XIST promotes the migration and invasion of papillary TC cells by modulating the miR-101-3p/CLDN1 axis [25]. This study explored the regulatory mechanism of XIST and HIF-1a in TC.

Biomarkers in serum have the advantages of convenient and noninvasive detection. XIST is stably expressed in serum and can be used as a tumor marker [26,27]. In this study, we first verified that HIF-1a is overexpressed in TC tissues, and its expression level is correlated with the expression level of XIST in serum. These results indicate that high expression of XIST in serum may represent the overexpression of HIF-1a in TC. Then, we verified the regulatory mechanism of XIST and HIF-1a: XIST serves as a ceRNA for miR-93-5p, competing with HIF-1a for miR-93-5p binding. Knockdown of XIST could inhibit the level of glycolysis in TC cells, and downregulation of miR-93 restored the level of glycolysis, proving that XIST regulates aerobic glycolysis levels through the XIST/miR-93/HIF-1a pathway. XIST could also regulate the proliferation, cell cycle and metastasis of TC cells. The cell cycle of TC cells was arrested in the G1 phase, and proliferation and metastasis were reduced by knockdown of XIST. The main function of the G1 phase is to synthesize RNA and ribosomes and prepare for DNA synthesis, which requires considerable energy and materials. Glycolysis is inhibited by knockdown of XIST, resulting in insufficient energy and material supply, which arrests the cell cycle in the G1 phase. These results explained the mechanism by which knockdown of XIST arrested the cell cycle in the G1 phase.

The high expression of XIST in serum could be used as a tumor marker reflecting that the progression of TC may be caused by active aerobic glycolysis. We found that artemisinin could target HIF-1a directly and inhibit the aerobic glycolysis promoted by HIF-1a overexpression. Artemisinin, an extract from the plant Artemisia annua, is a well-known and efficacious antimalarial drug. It has also been proven to be able to inhibit cancer [28,29]. We first proved that artemisinin could bind to HIF-1a spontaneously and promote the degradation of HIF-1a. Then, we proved that artemisinin could inhibit the glycolysis level, proliferation, cell cycle and metastasis of TC cells. These effects are consistent with those of XIST knockdown. Artemisinin may respond to the progression of thyroid cancer promoted by high expression of XIST.

In short, XIST serves as a ceRNA for miR-93, competing with HIF-1a for miR-93 binding, and finally promoting TC progression by regulating aerobic glycolysis. XIST levels in serum may be used as a tumor marker for TC promoted by HIF-1a, which could be treated by artemisinin.

## Supporting information

S1 Table Patient information of the TCGA database. (DOCX)

S2 Table Patient information of the Hebei Medical University Fourth Hospital. (DOCX)

S1 Raw images (PDF)

## References

[1] Haugen BR, Sawka AM, Alexander EK, Bible KC, Caturegli P, Doherty GM, et al. American Thyroid Association Guidelines on the Management of Thyroid Nodules and Differentiated Thyroid Cancer Task Force Review and Recommendation on the Proposed Renaming of Encapsulated Follicular Variant Papillary Thyroid Carcinoma Without Invasion to Noninvasive Follicular Thyroid Neoplasm with Papillary-Like Nuclear Features. Thyroid. 2017;27(4):481–3. doi: 10.1089/thy.2016.0628 28114862

[2] Sung H, Ferlay J, Siegel RL, Laversanne M, Soerjomataram I, Jemal A, et al. Global Cancer Statistics 2020: GLOBOCAN Estimates of Incidence and Mortality Worldwide for 36 Cancers in 185 Countries. CA Cancer J Clin. 2021;71(3):209–49. doi: 10.3322/caac.21660 33538338

[3] Siolek M, Cybulski C, Gasior-Perczak D, Kowalik A, Kozak-Klonowska B, Kowalska A, et al. CHEK2 mutations and the risk of papillary thyroid cancer. Int J Cancer. 2015;137(3):548–52. doi: 10.1002/ijc.29426 25583358

[4] Bellevicine C, Iaccarino A, Malapelle U, Sasso FC, Biondi B, Troncone G. PAX8 is expressed in anaplastic thyroid carcinoma diagnosed by fine-needle aspiration: a study of three cases with histological correlates. Eur J Endocrinol. 2013;169(3):307–11. doi: 10.1530/EJE-13-0150 23811186

[5] What are the risk factors for thyroid cancer?; 2016.

[6] Liu X, Bishop J, Shan Y, Pai S, Liu D, Murugan AK, et al. Highly prevalent TERT promoter mutations in aggressive thyroid cancers. Endocr Relat Cancer. 2013;20(4):603–10. doi: 10.1530/ERC-13-0210 23766237 PMC3782569

[7] Murugan AK, Xing M. Anaplastic thyroid cancers harbor novel oncogenic mutations of the ALK gene. Cancer Res. 2011;71(13):4403–11. doi: 10.1158/0008-5472.CAN-10-4041 21596819 PMC3129369

[8] Murugan AK, Bojdani E, Xing M. Identification and functional characterization of isocitrate dehydrogenase 1 (IDH1) mutations in thyroid cancer. Biochem Biophys Res Commun. 2010;393(3):555–9. doi: 10.1016/j.bbrc.2010.02.095 20171178 PMC2838977

[9] Murugan AK, Dong J, Xie J, Xing M. Uncommon GNAQ, MMP8, AKT3, EGFR, and PIK3R1 mutations in thyroid cancers. Endocr Pathol. 2011;22(2):97–102. doi: 10.1007/s12022-011-9155-x 21487925 PMC3133631

[10] Bose S, Le A. Glucose Metabolism in Cancer. Adv Exp Med Biol. 2018;1063:3–12. doi: 10.1007/978-3-319-77736-8_1 29946772

[11] Huang P, Chang S, Jiang X, Su J, Dong C, Liu X, et al. RNA interference targeting CD147 inhibits the proliferation, invasiveness, and metastatic activity of thyroid carcinoma cells by down-regulating glycolysis. Int J Clin Exp Pathol. 2015;8(1):309–18. 25755717 PMC4348865

[12] Nagao A, Kobayashi M, Koyasu S, Chow C, Harada H. HIF-1-Dependent Reprogramming of Glucose Metabolic Pathway of Cancer Cells and Its Therapeutic Significance. Int J Mol Sci. 2019;20(2). doi: 10.3390/ijms20020238 30634433 PMC6359724

[13] Jiang Y, Wu GH, He GD, Zhuang QL, Xi QL, Zhang B, et al. The Effect of Silencing HIF-1alpha Gene in BxPC-3 Cell Line on Glycolysis-Related Gene Expression, Cell Growth, Invasion, and Apoptosis. Nutr Cancer. 2015;67(8):1314–23. doi: http%3A//doi.org/10.1080/01635581.2015.1085584 26576476 10.1080/01635581.2015.1085584

[14] Yang LG, Cao MZ, Zhang J, Li XY, Sun QL. LncRNA XIST modulates HIF-1A/AXL signaling pathway by inhibiting miR-93-5p in colorectal cancer. Mol Genet Genomic Med. 2020;8(4):e1112. doi: 10.1002/mgg3.1112 32061057 PMC7196477

[15] Liu H, Deng H, Zhao Y, Li C, Liang Y. LncRNA XIST/miR-34a axis modulates the cell proliferation and tumor growth of thyroid cancer through MET-PI3K-AKT signaling. J Exp Clin Cancer Res. 2018;37(1):279. doi: 10.1186/s13046-018-0950-9 30463570 PMC6249781

[16] Brown CJ, Ballabio A, Rupert JL, Lafreniere RG, Grompe M, Tonlorenzi R, et al. A gene from the region of the human X inactivation centre is expressed exclusively from the inactive X chromosome. Nature. 1991;349(6304):38–44. doi: 10.1038/349038a0 1985261

[17] Murugan AK, Munirajan AK, Alzahrani AS. Long noncoding RNAs: emerging players in thyroid cancer pathogenesis. Endocr Relat Cancer. 2018;25(2):R59–82. doi: 10.1530/ERC-17-0188 29146581

[18] Guo D, Jin J, Liu J, Wang Y, Li D, He Y. Baicalein Inhibits the Progression and Promotes Radiosensitivity of Esophageal Squamous Cell Carcinoma by Targeting HIF-1A. Drug Des Devel Ther. 2022;16:2423–36. doi: 10.2147/DDDT.S370114 35937565 PMC9346416

[19] WARBURG O. On the origin of cancer cells. Science. 1956;123(3191):309–14. doi: 10.1126/science.123.3191.309 13298683

[20] Kobliakov VA. The Mechanisms of Regulation of Aerobic Glycolysis (Warburg Effect) by Oncoproteins in Carcinogenesis. Biochemistry (Mosc). 2019;84(10):1117–28. doi: 10.1134/S0006297919100018 31694508

[21] Vaupel P, Mayer A. Hypoxia in tumors: pathogenesis-related classification, characterization of hypoxia subtypes, and associated biological and clinical implications. Adv Exp Med Biol. 2014;812:19–24. doi: 10.1007/978-1-4939-0620-8_3 24729210

[22] Zhou J, Schmid T, Schnitzer S, Brune B. Tumor hypoxia and cancer progression. Cancer Lett. 2006;237(1):10–21. doi: 10.1016/j.canlet.2005.05.028 16002209

[23] Feldser D, Agani F, Iyer NV, Pak B, Ferreira G, Semenza GL. Reciprocal positive regulation of hypoxia-inducible factor 1alpha and insulin-like growth factor 2. Cancer Res. 1999;59(16):3915–8. 10463582

[24] Jiang BH, Agani F, Passaniti A, Semenza GL. V-SRC induces expression of hypoxia-inducible factor 1 (HIF-1) and transcription of genes encoding vascular endothelial growth factor and enolase 1: involvement of HIF-1 in tumor progression. Cancer Res. 1997;57(23):5328–35. 9393757

[25] Du YL, Liang Y, Cao Y, Liu L, Li J, Shi GQ. LncRNA XIST Promotes Migration and Invasion of Papillary Thyroid Cancer Cell by Modulating MiR-101-3p/CLDN1 Axis. Biochem Genet. 2021;59(2):437–52. doi: 10.1007/s10528-020-09985-8 33057875

[26] Yu J, Dong W, Liang J. Extracellular Vesicle-Transported Long Non-Coding RNA (LncRNA) X Inactive-Specific Transcript (XIST) in Serum is a Potential Novel Biomarker for Colorectal Cancer Diagnosis. Med Sci Monit. 2020;26:e924448. doi: 10.12659/MSM.924448 32843612 PMC7448689

[27] Lan F, Zhang X, Li H, Yue X, Sun Q. Serum exosomal lncRNA XIST is a potential non-invasive biomarker to diagnose recurrence of triple-negative breast cancer. J Cell Mol Med. 2021;25(16):7602–7. doi: 10.1111/jcmm.16009 33949761 PMC8358868

[28] Wong YK, Xu C, Kalesh KA, He Y, Lin Q, Wong W, et al. Artemisinin as an anticancer drug: Recent advances in target profiling and mechanisms of action. Med Res Rev. 2017;37(6):1492–517. doi: 10.1002/med.21446 28643446

[29] Kiani BH, Kayani WK, Khayam AU, Dilshad E, Ismail H, Mirza B. Artemisinin and its derivatives: a promising cancer therapy. Mol Biol Rep. 2020;47(8):6321–36. doi: 10.1007/s11033-020-05669-z 32710388

