# Supplementary material for: Artemisinin suppresses aerobic glycolysis in thyroid cancer cells by downregulating HIF-1a, which is increased by the XIST/miR-93/HIF-1a pathway
Source: PLoS One. 2023 Apr 10;18(4):e0284242. doi: 10.1371/journal.pone.0284242 (PMC10085032; doi:10.1371/journal.pone.0284242)
Supplement: S1 Table — (DOCX) [file pone.0284242.s001.docx]

**S1 Table. Patient information of the TCGA database.**

| ID | age | gender | stage | T | M | N | XIST | HIF1A |
| --- | --- | --- | --- | --- | --- | --- | --- | --- |
| TCGA-4C-A93U | ≥65 | FEMALE | Stage IV | T4 | M0 | N1b | 10.02275165 | 63.23069281 |
| TCGA-BJ-A0YZ | ≥65 | MALE | Stage II | T2 | M0 | N0 | 0.000879945 | 26.04680068 |
| TCGA-BJ-A0Z0 | <65 | MALE | Stage II | T2 | MX | N0 | 0.00975708 | 39.40780517 |
| TCGA-BJ-A0Z2 | <65 | MALE | Stage IV | T2 | M1 | N0 | 0.026696925 | 27.59339839 |
| TCGA-BJ-A0Z3 | <65 | FEMALE | Stage I | T1 | M0 | N0 | 3.496553327 | 40.02228035 |
| TCGA-BJ-A0Z5 | <65 | MALE | Stage III | T3 | M0 | N1a | 0.005401841 | 50.15336325 |
| TCGA-BJ-A0Z9 | <65 | FEMALE | Stage III | T3 | MX | N0 | 5.841831358 | 103.7037288 |
| TCGA-BJ-A0ZA | ≥65 | FEMALE | Stage II | T2 | M0 | N0 | 5.53313077 | 26.22270648 |
| TCGA-BJ-A0ZB | ≥65 | MALE | Stage IV | T3 | M0 | N1b | 0.007577175 | 43.57249003 |
| TCGA-BJ-A0ZC | <65 | MALE | Stage I | T1 | M0 | N0 | 0 | 37.33614375 |
| TCGA-BJ-A0ZE | <65 | FEMALE | Stage III | T3 | M0 | N0 | 1.094262572 | 16.44575823 |
| TCGA-BJ-A0ZF | <65 | FEMALE | Stage II | T2 | M0 | N0 | 0.758526423 | 24.99533441 |
| TCGA-BJ-A0ZG | ≥65 | MALE | Stage I | T1 | M0 | N0 | 0.004363334 | 28.94801587 |
| TCGA-BJ-A0ZH | <65 | FEMALE | Stage IV | T2 | MX | N1b | 9.091607816 | 39.05818251 |
| TCGA-BJ-A0ZJ | <65 | MALE | Stage I | T2 | M0 | N1a | 0.00294446 | 32.73246917 |
| TCGA-BJ-A18Y | <65 | MALE | Stage I | T1 | MX | N0 | 0.004972258 | 24.87973433 |
| TCGA-BJ-A18Z | <65 | MALE | Stage IV | T3 | MX | N1b | 0 | 51.20456056 |
| TCGA-BJ-A190 | <65 | MALE | Stage II | T2 | MX | N0 | 0.005553056 | 32.89344035 |
| TCGA-BJ-A191 | <65 | FEMALE | Stage I | T1 | M0 | N0 | 23.0983346 | 23.11351364 |
| TCGA-BJ-A28R | <65 | FEMALE | Stage I | T1 | M0 | N0 | 23.77865803 | 49.12778785 |
| TCGA-BJ-A28R | <65 | FEMALE | Stage I | T1 | M0 | N0 | 9.533075541 | 39.83170337 |
| TCGA-BJ-A28S | ≥65 | MALE | Stage II | T2 | M0 | N0 | 0.00105531 | 27.848519 |
| TCGA-BJ-A28T | <65 | FEMALE | Stage I | T1 | M0 | N1a | 22.13637274 | 38.60389333 |
| TCGA-BJ-A28V | ≥65 | FEMALE | Stage III | T3 | MX | N0 | 13.01588369 | 31.53642409 |
| TCGA-BJ-A28W | <65 | FEMALE | Stage I | T2 | M0 | N0 | 0.228566853 | 12.16982521 |
| TCGA-BJ-A28W | <65 | FEMALE | Stage I | T2 | M0 | N0 | 0.108371119 | 10.96096903 |
| TCGA-BJ-A28X | <65 | FEMALE | Stage I | T3 | M0 | N1a | 29.07500365 | 33.42404231 |
| TCGA-BJ-A28X | <65 | FEMALE | Stage I | T3 | M0 | N1a | 15.34824698 | 48.04344881 |
| TCGA-BJ-A28Z | <65 | FEMALE | Stage III | T3 | M0 | N1a | 4.650314348 | 66.73420562 |
| TCGA-BJ-A290 | ≥65 | MALE | Stage III | T3 | M0 | N1a | 0.008950502 | 39.20408771 |
| TCGA-BJ-A290 | ≥65 | MALE | Stage III | T3 | M0 | N1a | 0.01295611 | 26.95969758 |
| TCGA-BJ-A291 | <65 | FEMALE | Stage I | T1 | M0 | N0 | 0.415567447 | 25.80038816 |
| TCGA-BJ-A2N7 | <65 | FEMALE | Stage I | T3 | M0 | N0 | 3.171375404 | 24.67956239 |
| TCGA-BJ-A2N7 | <65 | FEMALE | Stage I | T3 | M0 | N0 | 17.88023381 | 34.46224806 |
| TCGA-BJ-A2N8 | <65 | FEMALE | Stage I | T1 | M0 | N0 | 7.499084557 | 37.00856902 |
| TCGA-BJ-A2N8 | <65 | FEMALE | Stage I | T1 | M0 | N0 | 3.012083431 | 24.60929286 |
| TCGA-BJ-A2N9 | <65 | FEMALE | Stage I | T2 | M0 | N0 | 4.574339256 | 25.96773712 |
| TCGA-BJ-A2N9 | <65 | FEMALE | Stage I | T2 | M0 | N0 | 0.335821007 | 14.71061434 |
| TCGA-BJ-A2NA | ≥65 | MALE | Stage III | T3 | M0 | N0 | 0.005011329 | 34.12351695 |
| TCGA-BJ-A2NA | ≥65 | MALE | Stage III | T3 | M0 | N0 | 0 | 63.19231015 |
| TCGA-BJ-A2P4 | <65 | FEMALE | Stage I | T2 | M0 | N0 | 1.666843015 | 16.78471788 |
| TCGA-BJ-A3EZ | <65 | MALE | Stage IV | T3 | M0 | N1b | 0.00299959 | 46.26099703 |
| TCGA-BJ-A3F0 | <65 | FEMALE | Stage I | T1 | M0 | N0 | 0.25232666 | 20.78252344 |
| TCGA-BJ-A3PR | ≥65 | FEMALE | Stage I | T1 | M0 | N0 | 4.807510642 | 45.84669893 |
| TCGA-BJ-A3PR | ≥65 | FEMALE | Stage I | T1 | M0 | N0 | 0.22052623 | 11.68214917 |
| TCGA-BJ-A3PT | <65 | FEMALE | Stage III | T3 | M0 | N0 | 0.867569248 | 27.52975237 |
| TCGA-BJ-A3PU | <65 | MALE | Stage III | T3 | M0 | N1a | 0.000657512 | 29.8732893 |
| TCGA-BJ-A3PU | <65 | MALE | Stage III | T3 | M0 | N1a | 0.010828954 | 39.34178455 |
| TCGA-BJ-A45C | ≥65 | MALE | Stage III | T3 | M0 | N0 | 0.001748288 | 13.77975967 |
| TCGA-BJ-A45D | <65 | MALE | Stage I | T2 | M0 | N0 | 0 | 17.22503755 |
| TCGA-BJ-A45E | <65 | FEMALE | Stage I | T1 | M0 | N0 | 11.21081583 | 28.48609573 |
| TCGA-BJ-A45F | <65 | FEMALE | Stage I | T1 | M0 | N0 | 15.47631796 | 17.4749384 |
| TCGA-BJ-A45G | <65 | FEMALE | Stage III | T2 | M0 | N1 | 18.934412 | 24.7179894 |
| TCGA-BJ-A45H | <65 | MALE | Stage III | T3 | M0 | N0 | 0.008117064 | 41.87827702 |
| TCGA-BJ-A45I | <65 | FEMALE | Stage III | T3 | M0 | N0 | 10.96197633 | 47.42131574 |
| TCGA-BJ-A45J | <65 | FEMALE | Stage I | T1 | M0 | N1a | 3.37656778 | 42.52652427 |
| TCGA-BJ-A45K | <65 | MALE | Stage I | T2 | M0 | N0 | 0.000793287 | 24.0894476 |
| TCGA-BJ-A4O8 | <65 | MALE | Stage III | T3 | M0 | N1a | 0.002650515 | 59.5791134 |
| TCGA-BJ-A4O9 | <65 | FEMALE | Stage II | T2 | M0 | N0 | 4.133768299 | 32.7402942 |
| TCGA-CE-A13K | <65 | FEMALE | Stage I | T3 | M0 | N1 | 11.71901585 | 47.82360869 |
| TCGA-CE-A27D | <65 | FEMALE | Stage I | T2 | M0 | N1 | 2.573292864 | 40.18298099 |
| TCGA-CE-A3MD | <65 | MALE | Stage I | T2 | M0 | N1 | 0.00616598 | 39.2878189 |
| TCGA-CE-A3ME | <65 | FEMALE | Stage II | T2 | M0 | N0 | 8.643486972 | 45.74778358 |
| TCGA-CE-A481 | <65 | FEMALE | Stage I | T1 | M0 | N0 | 18.32106017 | 39.37343725 |
| TCGA-CE-A482 | <65 | FEMALE | Stage I | T1 | M0 | N1 | 22.9059059 | 54.27809096 |
| TCGA-CE-A483 | <65 | FEMALE | Stage I | T2 | M0 | N1 | 23.02522734 | 44.62808421 |
| TCGA-CE-A484 | <65 | FEMALE | Stage I | T2 | M0 | N1 | 5.155275742 | 12.73350524 |
| TCGA-CE-A485 | <65 | MALE | Stage I | T2 | M0 | N1 | 0.0025457 | 47.66181043 |
| TCGA-DE-A0XZ | ≥65 | FEMALE | Stage III | T1 | M0 | N1a | 18.89228308 | 45.49443266 |
| TCGA-DE-A0Y2 | <65 | FEMALE | Stage I | T2 | M0 | N1a | 12.14731283 | 68.51415777 |
| TCGA-DE-A0Y3 | <65 | FEMALE | Stage IV | T4 | M1 | N1b | 6.641773403 | 41.80452738 |
| TCGA-DE-A3KN | <65 | FEMALE | Stage IV | T3 | M0 | N1b | 12.59797985 | 46.37291839 |
| TCGA-DE-A4M8 | <65 | FEMALE | Stage III | T2 | M0 | N1 | 10.17060248 | 40.62217089 |
| TCGA-DE-A4MA | <65 | FEMALE | Stage III | T3 | M0 | N1a | 0.153713945 | 15.64708932 |
| TCGA-DE-A4MB | ≥65 | FEMALE | Stage IV | T3 | M0 | N1b | 8.222100369 | 102.1784371 |
| TCGA-DE-A4MC | <65 | FEMALE | Stage I | T3 | M0 | N1b | 14.596868 | 92.03001244 |
| TCGA-DE-A4MD | ≥65 | MALE | Stage IV | T3 | M0 | N1b | 0.000844503 | 19.23353465 |
| TCGA-DE-A4MD | ≥65 | MALE | Stage IV | T3 | M0 | N1b | 0.001561385 | 25.26571709 |
| TCGA-DE-A69K | <65 | FEMALE | Stage III | T3 | M0 | N0 | 6.997606398 | 44.76318138 |
| TCGA-DE-A7U5 | <65 | FEMALE | Stage I | T2 | M0 | N0 | 10.83715731 | 56.79519153 |
| TCGA-DJ-A13L | ≥65 | MALE | Stage I | T1 | M0 | N0 | 0.008270054 | 62.61758941 |
| TCGA-DJ-A13M | <65 | FEMALE | Stage I | T1 | M0 | N0 | 0.370736956 | 19.66488685 |
| TCGA-DJ-A13O | <65 | MALE | Stage I | T1 | M0 | N0 | 0.001440561 | 34.69461051 |
| TCGA-DJ-A13P | <65 | FEMALE | Stage II | T2 | M0 | N0 | 9.813390994 | 52.34757832 |
| TCGA-DJ-A13R | <65 | MALE | Stage III | T3 | M0 | N0 | 0 | 9.07077079 |
| TCGA-DJ-A13S | <65 | FEMALE | Stage I | T1 | M0 | N0 | 5.939404135 | 39.11644924 |
| TCGA-DJ-A13T | <65 | FEMALE | Stage I | T2 | M0 | N0 | 6.188120342 | 47.00834708 |
| TCGA-DJ-A13U | <65 | MALE | Stage III | T3 | M0 | N0 | 0.001529088 | 42.41590026 |
| TCGA-DJ-A13V | <65 | FEMALE | Stage I | T3 | M0 | N1a | 10.50283603 | 46.64992173 |
| TCGA-DJ-A13W | <65 | FEMALE | Stage I | T1 | M0 | N0 | 0.240205027 | 6.65646083 |
| TCGA-DJ-A13X | <65 | FEMALE | Stage III | T3 | M0 | N0 | 12.13819584 | 44.91202309 |
| TCGA-DJ-A1QD | <65 | FEMALE | Stage I | T2 | M0 | N1b | 7.234681449 | 54.26810765 |
| TCGA-DJ-A1QE | <65 | FEMALE | Stage III | T3 | M0 | N0 | 3.69580458 | 69.17510712 |
| TCGA-DJ-A1QF | <65 | FEMALE | Stage II | T2 | M0 | N0 | 5.627717907 | 63.3695032 |
| TCGA-DJ-A1QG | <65 | MALE | Stage I | T1 | M0 | N0 | 0.02117805 | 34.61365135 |
| TCGA-DJ-A1QH | <65 | FEMALE | Stage III | T3 | M0 | N1a | 4.278357145 | 71.13251781 |
| TCGA-DJ-A1QI | <65 | FEMALE | Stage II | T2 | M0 | N0 | 9.211654297 | 87.11515663 |
| TCGA-DJ-A1QL | ≥65 | MALE | Stage II | T2 | M0 | N0 | 0.012265673 | 23.1225754 |
| TCGA-DJ-A1QM | <65 | MALE | Stage I | T1 | M0 | N0 | 0.007842682 | 49.10424312 |
| TCGA-DJ-A1QN | <65 | FEMALE | Stage I | T1 | M0 | N0 | 10.25961457 | 72.87649331 |
| TCGA-DJ-A1QO | ≥65 | MALE | Stage III | T3 | M0 | N0 | 0.007372011 | 46.74867682 |
| TCGA-DJ-A1QQ | <65 | MALE | Stage I | T3 | M0 | N0 | 0.00779467 | 32.9893078 |
| TCGA-DJ-A2PN | ≥65 | FEMALE | Stage I | T1 | M0 | N0 | 13.63981969 | 52.22791244 |
| TCGA-DJ-A2PO | <65 | MALE | Stage I | T1 | M0 | N0 | 0.001660522 | 54.19277797 |
| TCGA-DJ-A2PP | <65 | MALE | Stage II | T2 | M0 | N0 | 0.004746176 | 39.21365895 |
| TCGA-DJ-A2PQ | <65 | MALE | Stage I | T2 | M0 | N1a | 0.016948745 | 48.04532431 |
| TCGA-DJ-A2PR | <65 | MALE | Stage I | T3 | M0 | N1a | 0 | 42.12431275 |
| TCGA-DJ-A2PS | <65 | FEMALE | Stage I | T3 | M0 | N1a | 8.622409852 | 66.72968446 |
| TCGA-DJ-A2PT | ≥65 | FEMALE | Stage III | T3 | M0 | N0 | 0.188685957 | 54.44623158 |
| TCGA-DJ-A2PU | <65 | FEMALE | Stage II | T2 | M0 | N0 | 2.686100253 | 46.30586967 |
| TCGA-DJ-A2PV | <65 | FEMALE | Stage II | T2 | M0 | N0 | 9.394834809 | 48.36304814 |
| TCGA-DJ-A2PW | ≥65 | MALE | Stage III | T3 | M0 | N1a | 0.00303516 | 67.04989855 |
| TCGA-DJ-A2PX | <65 | FEMALE | Stage I | T1 | M0 | N0 | 8.266145882 | 42.59784301 |
| TCGA-DJ-A2PY | <65 | FEMALE | Stage III | T3 | M0 | N1a | 10.34468346 | 70.50497806 |
| TCGA-DJ-A2PZ | <65 | MALE | Stage III | T3 | M0 | N0 | 0.004784875 | 49.77764451 |
| TCGA-DJ-A2Q0 | <65 | FEMALE | Stage II | T2 | M0 | N0 | 5.768069888 | 32.16913074 |
| TCGA-DJ-A2Q1 | <65 | FEMALE | Stage I | T1 | M0 | N1b | 0.496221824 | 44.70591219 |
| TCGA-DJ-A2Q2 | <65 | FEMALE | Stage III | T3 | M0 | N0 | 9.091629629 | 41.34629174 |
| TCGA-DJ-A2Q3 | <65 | FEMALE | Stage III | T3 | M0 | N1a | 7.146867789 | 49.90404254 |
| TCGA-DJ-A2Q4 | <65 | MALE | Stage III | T1 | M0 | N1a | 0.611192878 | 50.53705756 |
| TCGA-DJ-A2Q5 | <65 | MALE | Stage IV | T2 | M0 | N1b | 0.738403026 | 66.15577308 |
| TCGA-DJ-A2Q6 | <65 | FEMALE | Stage I | T1 | M0 | N1b | 10.29480403 | 54.97636666 |
| TCGA-DJ-A2Q7 | <65 | FEMALE | Stage III | T2 | M0 | N1a | 3.340545764 | 43.67509002 |
| TCGA-DJ-A2Q9 | ≥65 | FEMALE | Stage III | T1 | M0 | N1a | 3.293618324 | 109.5222393 |
| TCGA-DJ-A2QA | <65 | FEMALE | Stage III | T3 | M0 | N0 | 0.020049538 | 7.776113436 |
| TCGA-DJ-A2QB | <65 | FEMALE | Stage IV | T3 | M0 | N1b | 2.407411295 | 34.47807134 |
| TCGA-DJ-A2QC | ≥65 | FEMALE | Stage II | T2 | M0 | N0 | 0.669423648 | 17.33380094 |
| TCGA-DJ-A3UK | <65 | FEMALE | Stage I | T1 | M0 | N0 | 25.36548742 | 43.71203311 |
| TCGA-DJ-A3UM | <65 | FEMALE | Stage I | T1 | M0 | N0 | 8.519185446 | 46.36326201 |
| TCGA-DJ-A3UN | <65 | FEMALE | Stage I | T1 | M0 | N0 | 10.63531088 | 26.7642993 |
| TCGA-DJ-A3UO | <65 | MALE | Stage IV | T3 | M0 | N1b | 0.01261909 | 67.95943556 |
| TCGA-DJ-A3UP | <65 | FEMALE | Stage I | T2 | M0 | N0 | 27.84725704 | 36.34920461 |
| TCGA-DJ-A3UQ | <65 | FEMALE | Stage III | T3 | M0 | N1a | 10.19986751 | 59.03781207 |
| TCGA-DJ-A3UR | <65 | FEMALE | Stage III | T3 | M0 | N1a | 7.862026141 | 36.8660029 |
| TCGA-DJ-A3US | <65 | FEMALE | Stage I | T1 | M0 | N0 | 14.09006498 | 45.10128753 |
| TCGA-DJ-A3UT | <65 | FEMALE | Stage I | T1 | M0 | N0 | 12.94877065 | 22.79379692 |
| TCGA-DJ-A3UU | <65 | FEMALE | Stage III | T3 | M0 | N0 | 17.96419436 | 38.85007715 |
| TCGA-DJ-A3UV | <65 | FEMALE | Stage I | T1 | M0 | N0 | 12.91328292 | 48.33233343 |
| TCGA-DJ-A3UW | <65 | FEMALE | Stage I | T1 | M0 | N0 | 7.966774728 | 59.19365105 |
| TCGA-DJ-A3UX | <65 | FEMALE | Stage I | T1 | M0 | N0 | 16.5077627 | 32.7463571 |
| TCGA-DJ-A3UY | <65 | FEMALE | Stage I | T1 | M0 | N0 | 9.396076304 | 53.10008613 |
| TCGA-DJ-A3UZ | ≥65 | FEMALE | Stage III | T3 | M0 | N0 | 19.90171062 | 33.81425534 |
| TCGA-DJ-A3V0 | <65 | MALE | Stage IV | T4 | M0 | N1b | 0.003869966 | 29.59508403 |
| TCGA-DJ-A3V2 | <65 | FEMALE | Stage I | T2 | M0 | N1a | 3.853224267 | 42.63871935 |
| TCGA-DJ-A3V3 | <65 | FEMALE | Stage II | T2 | M0 | N0 | 7.184999033 | 36.10403713 |
| TCGA-DJ-A3V4 | <65 | FEMALE | Stage III | T3 | M0 | N1a | 16.080578 | 51.07902147 |
| TCGA-DJ-A3V5 | ≥65 | FEMALE | Stage III | T3 | M0 | N0 | 13.79493743 | 48.96021461 |
| TCGA-DJ-A3V6 | <65 | MALE | Stage III | T3 | M0 | N0 | 0.006134433 | 48.11675738 |
| TCGA-DJ-A3V7 | <65 | FEMALE | Stage III | T3 | M0 | N0 | 6.012012001 | 41.35971633 |
| TCGA-DJ-A3V8 | <65 | FEMALE | Stage I | T3 | M0 | N1b | 8.257797341 | 38.54291065 |
| TCGA-DJ-A3V9 | <65 | FEMALE | Stage IV | T2 | M0 | N1b | 14.73951794 | 48.24276497 |
| TCGA-DJ-A3VA | <65 | FEMALE | Stage I | T1 | M0 | N1a | 17.12943277 | 59.34644438 |
| TCGA-DJ-A3VB | <65 | MALE | Stage III | T3 | M0 | N1a | 0.001011053 | 52.00174696 |
| TCGA-DJ-A3VD | <65 | FEMALE | Stage I | T1 | M0 | N1a | 16.17773565 | 57.85528359 |
| TCGA-DJ-A3VE | <65 | MALE | Stage I | T1 | M0 | N0 | 0.009258566 | 42.16554505 |
| TCGA-DJ-A3VF | <65 | FEMALE | Stage IV | T3 | M0 | N1b | 11.03633857 | 61.56622195 |
| TCGA-DJ-A3VG | <65 | MALE | Stage I | T1 | M0 | N0 | 0.004953535 | 20.26219508 |
| TCGA-DJ-A3VI | <65 | FEMALE | Stage III | T1 | M0 | N1a | 12.55338972 | 58.49039722 |
| TCGA-DJ-A3VJ | <65 | MALE | Stage I | T3 | M0 | N1a | 0.010677589 | 79.04715599 |
| TCGA-DJ-A3VK | <65 | MALE | Stage III | T3 | M0 | N0 | 0.01536576 | 38.22358712 |
| TCGA-DJ-A3VL | <65 | MALE | Stage I | T1 | M0 | N0 | 0.014037856 | 21.39714327 |
| TCGA-DJ-A3VM | ≥65 | FEMALE | Stage II | T2 | M0 | N0 | 13.00714523 | 28.23129392 |
| TCGA-DJ-A4UL | ≥65 | FEMALE | Stage I | T1 | M0 | N0 | 0.783570556 | 26.12522085 |
| TCGA-DJ-A4UP | <65 | FEMALE | Stage I | T1 | M0 | N1b | 7.159539596 | 38.85599599 |
| TCGA-DJ-A4UQ | <65 | MALE | Stage IV | T3 | M0 | N1b | 0.117300281 | 32.63004539 |
| TCGA-DJ-A4UR | <65 | FEMALE | Stage II | T3 | M1 | N1b | 14.18879491 | 17.65333257 |
| TCGA-DJ-A4UT | <65 | FEMALE | Stage I | T1 | M0 | N0 | 11.83267113 | 60.35581271 |
| TCGA-DJ-A4UW | <65 | FEMALE | Stage I | T1 | M0 | N1b | 12.88604325 | 37.48614615 |
| TCGA-DJ-A4V0 | <65 | FEMALE | Stage I | T2 | M0 | N0 | 18.34565274 | 34.57751148 |
| TCGA-DJ-A4V2 | <65 | FEMALE | Stage I | T2 | M0 | N0 | 16.38545151 | 63.37961577 |
| TCGA-DJ-A4V4 | <65 | FEMALE | Stage I | T1 | M0 | N0 | 8.497975679 | 39.36591064 |
| TCGA-DJ-A4V5 | <65 | MALE | Stage IV | T3 | M0 | N1b | 0.005000956 | 64.25954818 |
| TCGA-DO-A1K0 | <65 | FEMALE | Stage I | T3 | MX | N1b | 23.97898692 | 68.06258734 |
| TCGA-DO-A2HM | <65 | MALE | Stage IV | T1 | MX | N1b | 0.004025386 | 53.92588141 |
| TCGA-E3-A3DY | <65 | MALE | Stage I | T3 | M0 | N1a | 0.01313995 | 50.65301576 |
| TCGA-E3-A3DZ | <65 | FEMALE | Stage III | T3 | M0 | N0 | 13.35736181 | 35.71399419 |
| TCGA-E3-A3E0 | <65 | FEMALE | Stage I | T2 | MX | N0 | 9.917492671 | 43.34817739 |
| TCGA-E3-A3E1 | <65 | FEMALE | Stage I | T1 | M0 | N0 | 1.632266879 | 50.51358149 |
| TCGA-E3-A3E2 | <65 | FEMALE | Stage I | T1 | MX | N1a | 6.029290761 | 38.83853995 |
| TCGA-E3-A3E3 | <65 | FEMALE | Stage II | T2 | M0 | N0 | 5.379369888 | 45.28798354 |
| TCGA-E3-A3E5 | <65 | MALE | Stage IV | T4 | MX | N1b | 0.001743434 | 36.50570973 |
| TCGA-E8-A242 | <65 | FEMALE | Stage III | T3 | MX | N0 | 0.852961776 | 71.89104558 |
| TCGA-E8-A2EA | <65 | FEMALE | Stage I | T1 | M0 | N0 | 0.722147514 | 30.16386997 |
| TCGA-E8-A2JQ | <65 | FEMALE | Stage I | T3 | M0 | N1a | 4.015805467 | 39.01067956 |
| TCGA-E8-A2JQ | <65 | FEMALE | Stage I | T3 | M0 | N1a | 11.45848729 | 45.28359415 |
| TCGA-E8-A3X7 | <65 | FEMALE | Stage IV | T4 | M0 | N0 | 1.00546699 | 20.93224937 |
| TCGA-E8-A413 | <65 | FEMALE | Stage I | T1 | M0 | N0 | 8.648925682 | 47.57315784 |
| TCGA-E8-A414 | <65 | FEMALE | Stage III | T3 | M0 | N0 | 13.40228303 | 62.88152026 |
| TCGA-E8-A415 | <65 | FEMALE | Stage I | T1 | M0 | N1b | 16.5527226 | 46.84154035 |
| TCGA-E8-A416 | <65 | FEMALE | Stage I | T1 | M0 | N0 | 19.12215285 | 37.72396282 |
| TCGA-E8-A417 | <65 | FEMALE | Stage I | T2 | M0 | N1a | 21.37037992 | 38.80009731 |
| TCGA-E8-A418 | ≥65 | FEMALE | Stage IV | T4 | M0 | N0 | 14.55704615 | 130.2085161 |
| TCGA-E8-A419 | <65 | FEMALE | Stage I | T2 | M0 | N1 | 13.37620991 | 45.17289496 |
| TCGA-E8-A432 | <65 | FEMALE | Stage II | T2 | M0 | N0 | 3.743351155 | 49.97726815 |
| TCGA-E8-A433 | <65 | FEMALE | Stage I | T2 | M0 | N0 | 0.226017742 | 18.91379744 |
| TCGA-E8-A434 | <65 | FEMALE | Stage I | T1 | M0 | N0 | 3.835124356 | 33.61438096 |
| TCGA-E8-A436 | <65 | FEMALE | Stage IV | T2 | M0 | N1b | 9.529295249 | 53.35922329 |
| TCGA-E8-A438 | <65 | FEMALE | Stage I | T1 | M0 | N0 | 6.71544538 | 16.9132196 |
| TCGA-E8-A44K | <65 | FEMALE | Stage I | T1 | M0 | N0 | 7.700816768 | 26.6615075 |
| TCGA-E8-A44M | <65 | FEMALE | Stage I | T1 | M0 | N0 | 16.01662855 | 55.15700224 |
| TCGA-EL-A3CM | <65 | FEMALE | Stage III | T3 | M0 | N1b | 8.350958213 | 54.68695335 |
| TCGA-EL-A3CN | <65 | FEMALE | Stage II | T3 | M0 | N0 | 2.376697704 | 63.43179983 |
| TCGA-EL-A3CO | ≥65 | MALE | Stage III | T3 | M0 | N1b | 0.000803018 | 90.62631438 |
| TCGA-EL-A3CR | ≥65 | FEMALE | Stage III | T3 | M0 | N1b | 6.204693026 | 31.8637442 |
| TCGA-EL-A3CS | <65 | FEMALE | Stage IV | T4 | M0 | N0 | 2.143601403 | 60.37625582 |
| TCGA-EL-A3CT | ≥65 | FEMALE | Stage IV | T4 | M0 | N1a | 2.602608729 | 79.64443148 |
| TCGA-EL-A3CU | ≥65 | FEMALE | Stage III | T1 | M0 | N1a | 7.106323996 | 77.81341142 |
| TCGA-EL-A3CV | <65 | MALE | Stage I | T3 | M0 | N1b | 0.003655541 | 113.4308358 |
| TCGA-EL-A3CW | ≥65 | FEMALE | Stage III | T2 | M0 | N1a | 9.422088532 | 43.44397206 |
| TCGA-EL-A3CX | <65 | FEMALE | Stage I | T2 | M0 | N0 | 1.048569336 | 15.62407667 |
| TCGA-EL-A3CY | <65 | MALE | Stage I | T4 | M0 | N1b | 0.00226428 | 71.76949986 |
| TCGA-EL-A3CZ | <65 | FEMALE | Stage I | T1 | M0 | N1a | 10.68512501 | 39.77464629 |
| TCGA-EL-A3D0 | <65 | MALE | Stage III | T4 | M0 | N1b | 0.026839232 | 26.86458059 |
| TCGA-EL-A3D1 | <65 | MALE | Stage I | T2 | M0 | N0 | 0.006834465 | 13.12659052 |
| TCGA-EL-A3D4 | <65 | MALE | Stage IV | T3 | M0 | N1b | 0.002913155 | 17.44530697 |
| TCGA-EL-A3D5 | <65 | FEMALE | Stage I | T3 | M0 | N1b | 2.246057516 | 60.54624756 |
| TCGA-EL-A3D6 | <65 | FEMALE | Stage III | T3 | M0 | N1b | 7.884027863 | 67.74340935 |
| TCGA-EL-A3GO | <65 | FEMALE | Stage I | T2 | M0 | N0 | 19.7154057 | 33.47598118 |
| TCGA-EL-A3GP | ≥65 | MALE | Stage III | T3 | M0 | N1a | 0.001602991 | 46.61198596 |
| TCGA-EL-A3GQ | ≥65 | FEMALE | Stage I | T1 | M0 | N0 | 21.67329098 | 39.04114909 |
| TCGA-EL-A3GS | <65 | FEMALE | Stage I | T3 | M0 | N1 | 2.629049219 | 69.09264325 |
| TCGA-EL-A3GU | ≥65 | FEMALE | Stage III | T3 | M0 | N1 | 7.32885266 | 54.6375159 |
| TCGA-EL-A3GV | ≥65 | FEMALE | Stage III | T3 | M0 | N0 | 3.926194636 | 76.04029458 |
| TCGA-EL-A3GW | <65 | FEMALE | Stage I | T2 | M0 | N0 | 1.812895771 | 67.62693039 |
| TCGA-EL-A3GX | <65 | FEMALE | Stage I | T2 | M0 | N1 | 3.567313131 | 69.31329707 |
| TCGA-EL-A3GY | <65 | FEMALE | Stage II | T1 | M1 | N1 | 5.831288797 | 80.25480892 |
| TCGA-EL-A3GZ | <65 | FEMALE | Stage I | T1 | M0 | N0 | 6.921012949 | 27.54030157 |
| TCGA-EL-A3GZ | <65 | FEMALE | Stage I | T1 | M0 | N0 | 13.09399099 | 95.43603338 |
| TCGA-EL-A3H1 | ≥65 | FEMALE | Stage I | T1 | M0 | N0 | 5.089569515 | 31.69584896 |
| TCGA-EL-A3H1 | ≥65 | FEMALE | Stage I | T1 | M0 | N0 | 14.15037442 | 33.28957593 |
| TCGA-EL-A3H2 | <65 | MALE | Stage II | T2 | MX | N0 | 0.005350565 | 47.24466967 |
| TCGA-EL-A3H2 | <65 | MALE | Stage II | T2 | MX | N0 | 0.001591299 | 46.17656502 |
| TCGA-EL-A3H3 | <65 | FEMALE | Stage I | T2 | M0 | N1 | 2.130368525 | 116.6720281 |
| TCGA-EL-A3H4 | <65 | FEMALE | Stage IV | T4 | M0 | N1 | 2.175798433 | 54.93384597 |
| TCGA-EL-A3H5 | <65 | FEMALE | Stage IV | T4 | M0 | N1 | 2.886531206 | 32.12611691 |
| TCGA-EL-A3H7 | <65 | FEMALE | Stage I | T3 | M0 | N1 | 10.30972367 | 46.17814855 |
| TCGA-EL-A3H7 | <65 | FEMALE | Stage I | T3 | M0 | N1 | 10.88564088 | 82.54719182 |
| TCGA-EL-A3H8 | <65 | FEMALE | Stage I | T3 | M0 | N1 | 13.44620593 | 64.86902212 |
| TCGA-EL-A3MW | <65 | FEMALE | Stage II | T2 | M0 | N0 | 10.00110204 | 45.01367878 |
| TCGA-EL-A3MW | <65 | FEMALE | Stage II | T2 | M0 | N0 | 12.46196147 | 36.2929801 |
| TCGA-EL-A3MX | ≥65 | FEMALE | Stage IV | T4 | M1 | N1 | 11.17036969 | 20.28278572 |
| TCGA-EL-A3MX | ≥65 | FEMALE | Stage IV | T4 | M1 | N1 | 12.79234389 | 30.79605311 |
| TCGA-EL-A3MZ | ≥65 | MALE | Stage III | T4 | MX | N1 | 0 | 56.9996479 |
| TCGA-EL-A3N2 | <65 | FEMALE | Stage I | T2 | M0 | N1 | 10.70298039 | 34.12422225 |
| TCGA-EL-A3N2 | <65 | FEMALE | Stage I | T2 | M0 | N1 | 22.15438101 | 72.07215096 |
| TCGA-EL-A3T0 | <65 | FEMALE | Stage III | T3 | M0 | N1 | 16.41607414 | 28.95549858 |
| TCGA-EL-A3T0 | <65 | FEMALE | Stage III | T3 | M0 | N1 | 10.14151667 | 47.43927883 |
| TCGA-EL-A3T1 | <65 | FEMALE | Stage I | T2 | M0 | N0 | 16.27521369 | 63.86295317 |
| TCGA-EL-A3T1 | <65 | FEMALE | Stage I | T2 | M0 | N0 | 12.60963622 | 74.23288027 |
| TCGA-EL-A3T2 | <65 | FEMALE | Stage III | T2 | M0 | N1 | 13.98058857 | 25.28719987 |
| TCGA-EL-A3T2 | <65 | FEMALE | Stage III | T2 | M0 | N1 | 21.83383312 | 32.75934914 |
| TCGA-EL-A3T3 | <65 | MALE | Stage II | T2 | M0 | N0 | 17.02295894 | 22.37606225 |
| TCGA-EL-A3T3 | <65 | MALE | Stage II | T2 | M0 | N0 | 6.268703148 | 30.32523974 |
| TCGA-EL-A3T6 | <65 | FEMALE | Stage I | T3 | M0 | N0 | 21.47758474 | 54.61385564 |
| TCGA-EL-A3T6 | <65 | FEMALE | Stage I | T3 | M0 | N0 | 11.15909878 | 70.73538607 |
| TCGA-EL-A3T7 | <65 | FEMALE | Stage II | T2 | M0 | N0 | 20.93807589 | 31.1118678 |
| TCGA-EL-A3T7 | <65 | FEMALE | Stage II | T2 | M0 | N0 | 7.88081207 | 56.74046424 |
| TCGA-EL-A3T8 | <65 | MALE | Stage I | T2 | M0 | N0 | 0.01098819 | 27.56626947 |
| TCGA-EL-A3T8 | <65 | MALE | Stage I | T2 | M0 | N0 | 0.005807577 | 34.35574686 |
| TCGA-EL-A3T9 | ≥65 | FEMALE | Stage IV | T4 | MX | N1 | 9.272888585 | 51.84255182 |
| TCGA-EL-A3TA | <65 | MALE | Stage I | T2 | M0 | N0 | 0.001333747 | 27.88599784 |
| TCGA-EL-A3TA | <65 | MALE | Stage I | T2 | M0 | N0 | 0.00164208 | 25.83445222 |
| TCGA-EL-A3TB | <65 | FEMALE | Stage III | T3 | M0 | N1a | 1.450357425 | 26.23887173 |
| TCGA-EL-A3TB | <65 | FEMALE | Stage III | T3 | M0 | N1a | 6.879199744 | 33.71918133 |
| TCGA-EL-A3ZG | <65 | MALE | Stage I | T3 | M0 | N0 | 0.126977997 | 15.23097502 |
| TCGA-EL-A3ZG | <65 | MALE | Stage I | T3 | M0 | N0 | 0.018598672 | 40.99855117 |
| TCGA-EL-A3ZH | <65 | FEMALE | Stage I | T3 | M0 | N1 | 42.31273898 | 34.47351237 |
| TCGA-EL-A3ZH | <65 | FEMALE | Stage I | T3 | M0 | N1 | 33.84906869 | 38.93149814 |
| TCGA-EL-A3ZK | <65 | FEMALE | Stage I | T2 | M0 | N1 | 16.45594099 | 48.79603656 |
| TCGA-EL-A3ZK | <65 | FEMALE | Stage I | T2 | M0 | N1 | 24.93807416 | 31.521537 |
| TCGA-EL-A3ZL | <65 | FEMALE | Stage I | T1 | M0 | N1 | 16.18417292 | 21.58167673 |
| TCGA-EL-A3ZL | <65 | FEMALE | Stage I | T1 | M0 | N1 | 23.54641012 | 34.95640204 |
| TCGA-EL-A3ZM | <65 | MALE | Stage IV | T4 | M0 | N1 | 0.006654191 | 21.67670805 |
| TCGA-EL-A3ZM | <65 | MALE | Stage IV | T4 | M0 | N1 | 0 | 25.07408838 |
| TCGA-EL-A3ZN | <65 | FEMALE | Stage I | T3 | M0 | N1 | 22.53812754 | 31.87897242 |
| TCGA-EL-A3ZO | ≥65 | FEMALE | Stage III | T3 | M0 | N1 | 1.463673467 | 42.14929904 |
| TCGA-EL-A3ZO | ≥65 | FEMALE | Stage III | T3 | M0 | N1 | 27.46450083 | 41.84675576 |
| TCGA-EL-A3ZP | <65 | MALE | Stage I | T4 | M0 | N1 | 0.012438162 | 36.42224199 |
| TCGA-EL-A3ZP | <65 | MALE | Stage I | T4 | M0 | N1 | 0 | 42.98569868 |
| TCGA-EL-A3ZQ | ≥65 | FEMALE | Stage II | T2 | M0 | N0 | 17.79388306 | 40.0855681 |
| TCGA-EL-A3ZQ | ≥65 | FEMALE | Stage II | T2 | M0 | N0 | 0.199676089 | 20.12119134 |
| TCGA-EL-A3ZR | <65 | FEMALE | Stage II | T2 | M0 | N0 | 12.25530374 | 33.21588663 |
| TCGA-EL-A3ZR | <65 | FEMALE | Stage II | T2 | M0 | N0 | 28.41726296 | 42.13240462 |
| TCGA-EL-A3ZS | <65 | FEMALE | Stage I | T1 | M0 | N1 | 0.250379224 | 24.13551656 |
| TCGA-EL-A3ZS | <65 | FEMALE | Stage I | T1 | M0 | N1 | 27.64327706 | 48.79074085 |
| TCGA-EL-A3ZT | <65 | MALE | Stage I | T3 | M0 | N0 | 0.000862694 | 25.8900006 |
| TCGA-EL-A3ZT | <65 | MALE | Stage I | T3 | M0 | N0 | 0.02046963 | 32.47976409 |
| TCGA-EL-A4JV | <65 | FEMALE | Stage I | T2 | M0 | N0 | 16.04930332 | 24.81657241 |
| TCGA-EL-A4JW | <65 | FEMALE | Stage I | T1 | M0 | N0 | 9.034252832 | 51.49236216 |
| TCGA-EL-A4JX | <65 | FEMALE | Stage I | T3 | MX | N0 | 9.066598785 | 55.89484824 |
| TCGA-EL-A4JZ | <65 | FEMALE | Stage III | T3 | M0 | N1a | 9.344712073 | 58.95616048 |
| TCGA-EL-A4K0 | <65 | FEMALE | Stage I | T1 | M0 | N0 | 15.54760278 | 75.56773141 |
| TCGA-EL-A4K1 | ≥65 | FEMALE | Stage III | T3 | M0 | N1 | 6.959750216 | 32.2494874 |
| TCGA-EL-A4K2 | <65 | FEMALE | Stage I | T1 | M0 | N0 | 8.51809414 | 27.48366686 |
| TCGA-EL-A4K4 | <65 | FEMALE | Stage I | T3 | M0 | N1 | 2.882741468 | 57.14748104 |
| TCGA-EL-A4K6 | ≥65 | MALE | Stage IV | T4 | MX | N1 | 0.006183811 | 11.50305172 |
| TCGA-EL-A4K7 | ≥65 | MALE | Stage III | T2 | M0 | N1 | 0.007384155 | 24.61941101 |
| TCGA-EL-A4K9 | ≥65 | MALE | Stage II | T2 | M0 | N0 | 0.006782306 | 44.86442929 |
| TCGA-EL-A4KD | <65 | MALE | Stage I | T3 | M0 | N1 | 0.001427633 | 49.84863133 |
| TCGA-EL-A4KG | <65 | FEMALE | Stage I | T3 | M0 | N0 | 1.65456876 | 61.72747622 |
| TCGA-EL-A4KH | <65 | FEMALE | Stage I | T2 | M0 | N0 | 0.167378488 | 17.40947361 |
| TCGA-EL-A4KI | <65 | MALE | Stage III | T3 | MX | N0 | 0.010219893 | 14.83989785 |
| TCGA-EM-A1CS | <65 | FEMALE | Stage I | T1 | MX | N0 | 6.662901819 | 30.62989056 |
| TCGA-EM-A1CS | <65 | FEMALE | Stage I | T1 | MX | N0 | 14.53852268 | 62.40562492 |
| TCGA-EM-A1CT | ≥65 | MALE | Stage IV | T1 | MX | N1b | 0.019950501 | 28.91794342 |
| TCGA-EM-A1CT | ≥65 | MALE | Stage IV | T1 | MX | N1b | 0.001375613 | 46.50993466 |
| TCGA-EM-A1CU | <65 | MALE | Stage I | T3 | M0 | N1a | 0.002784102 | 34.18881544 |
| TCGA-EM-A1CU | <65 | MALE | Stage I | T3 | M0 | N1a | 0.000815353 | 43.29443849 |
| TCGA-EM-A1CV | <65 | FEMALE | Stage I | T1 | MX | N0 | 4.524781782 | 24.46543618 |
| TCGA-EM-A1CV | <65 | FEMALE | Stage I | T1 | MX | N0 | 16.90958108 | 39.72814229 |
| TCGA-EM-A1CW | <65 | FEMALE | Stage I | T3 | MX | N0 | 0.655392409 | 24.07050485 |
| TCGA-EM-A1CW | <65 | FEMALE | Stage I | T3 | MX | N0 | 8.276512524 | 39.1963299 |
| TCGA-EM-A1YC | ≥65 | FEMALE | Stage III | T3 | MX | N0 | 6.014604869 | 20.7254645 |
| TCGA-EM-A1YC | ≥65 | FEMALE | Stage III | T3 | MX | N0 | 3.274077385 | 17.49768605 |
| TCGA-EM-A1YD | <65 | FEMALE | Stage II | T2 | MX | N0 | 6.639550689 | 51.6804064 |
| TCGA-EM-A1YE | <65 | FEMALE | Stage II | T2 | MX | N0 | 6.650889757 | 22.56664073 |
| TCGA-EM-A22I | <65 | FEMALE | Stage III | T3 | MX | N0 | 8.967581934 | 48.59855615 |
| TCGA-EM-A22J | <65 | FEMALE | Stage II | T2 | MX | N0 | 9.805239512 | 34.16284172 |
| TCGA-EM-A22K | <65 | FEMALE | Stage IV | T1 | MX | N1b | 19.39715504 | 38.59765721 |
| TCGA-EM-A22L | <65 | FEMALE | Stage I | T2 | MX | N0 | 1.039896638 | 29.67175576 |
| TCGA-EM-A22M | <65 | MALE | Stage I | T1 | MX | N0 | 0 | 40.84052897 |
| TCGA-EM-A22N | ≥65 | FEMALE | Stage I | T1 | MX | N0 | 17.01788622 | 33.06362256 |
| TCGA-EM-A22O | ≥65 | MALE | Stage IV | T4 | MX | N1a | 0 | 52.42274468 |
| TCGA-EM-A22P | <65 | MALE | Stage IV | T1 | MX | N1b | 0.005542081 | 36.16415568 |
| TCGA-EM-A22Q | <65 | MALE | Stage I | T3 | MX | N0 | 0.002623708 | 4.933888237 |
| TCGA-EM-A2CK | <65 | MALE | Stage I | T2 | MX | N1b | 0.008560647 | 27.71825112 |
| TCGA-EM-A2CM | <65 | FEMALE | Stage III | T3 | MX | N0 | 11.4543872 | 26.98212578 |
| TCGA-EM-A2CN | <65 | MALE | Stage IV | T3 | M1 | N0 | 0.005582517 | 17.58604761 |
| TCGA-EM-A2CP | <65 | FEMALE | Stage I | T2 | MX | N0 | 6.837947086 | 40.46554262 |
| TCGA-EM-A2CQ | <65 | FEMALE | Stage I | T1 | MX | N1a | 5.869427709 | 27.49519376 |
| TCGA-EM-A2CR | <65 | FEMALE | Stage II | T2 | MX | N0 | 0.232467467 | 7.028389163 |
| TCGA-EM-A2CS | <65 | FEMALE | Stage IV | T3 | MX | N1b | 9.93788623 | 35.39704451 |
| TCGA-EM-A2CS | <65 | FEMALE | Stage IV | T3 | MX | N1b | 0.66817883 | 20.24536942 |
| TCGA-EM-A2CT | <65 | FEMALE | Stage I | T1 | MX | N0 | 6.434368716 | 24.69902255 |
| TCGA-EM-A2CU | <65 | FEMALE | Stage III | T2 | MX | N1a | 1.673486592 | 32.78570011 |
| TCGA-EM-A2OV | <65 | FEMALE | Stage II | T2 | MX | N0 | 6.49296871 | 34.15558242 |
| TCGA-EM-A2OX | <65 | MALE | Stage IV | T1 | MX | N1b | 0.007665362 | 71.38816901 |
| TCGA-EM-A2OY | <65 | FEMALE | Stage II | T2 | MX | N0 | 14.00518618 | 27.9490993 |
| TCGA-EM-A2OZ | ≥65 | MALE | Stage I | T1 | MX | N0 | 0.00099659 | 46.16391365 |
| TCGA-EM-A2P0 | <65 | MALE | Stage I | T2 | MX | N1b | 0.007271414 | 49.60308874 |
| TCGA-EM-A2P1 | <65 | MALE | Stage I | T2 | MX | N1b | 0.016184062 | 70.0110946 |
| TCGA-EM-A2P1 | <65 | MALE | Stage I | T2 | MX | N1b | 0.000898246 | 49.89259679 |
| TCGA-EM-A2P2 | <65 | MALE | Stage III | T3 | MX | N0 | 0.002056304 | 42.41372667 |
| TCGA-EM-A3AJ | <65 | MALE | Stage I | T3 | MX | N1a | 0.003723085 | 34.49493286 |
| TCGA-EM-A3AK | <65 | FEMALE | Stage I | T1 | MX | N0 | 5.352904142 | 43.54458422 |
| TCGA-EM-A3AL | <65 | FEMALE | Stage I | T1 | MX | N0 | 8.087427779 | 34.15082193 |
| TCGA-EM-A3AN | <65 | FEMALE | Stage I | T1 | MX | N1b | 8.451165715 | 56.00043073 |
| TCGA-EM-A3AO | <65 | MALE | Stage IV | T2 | MX | N1b | 0.0034545 | 18.38583466 |
| TCGA-EM-A3AQ | ≥65 | FEMALE | Stage I | T1 | MX | N0 | 0.126305738 | 22.14658592 |
| TCGA-EM-A3AR | <65 | MALE | Stage I | T1 | MX | N1a | 0 | 33.2903267 |
| TCGA-EM-A3FJ | <65 | FEMALE | Stage I | T1 | MX | N1b | 5.696286998 | 43.56073361 |
| TCGA-EM-A3FK | <65 | FEMALE | Stage I | T1 | MX | N1b | 3.229729889 | 81.39451651 |
| TCGA-EM-A3FM | <65 | MALE | Stage IV | T2 | MX | N1b | 0.006203856 | 33.44677596 |
| TCGA-EM-A3FN | <65 | FEMALE | Stage I | T3 | MX | N0 | 0.323862489 | 16.34116153 |
| TCGA-EM-A3FO | <65 | MALE | Stage I | T3 | MX | N0 | 0 | 26.98957946 |
| TCGA-EM-A3FP | ≥65 | FEMALE | Stage II | T2 | MX | N0 | 0.460279985 | 18.63927984 |
| TCGA-EM-A3FQ | <65 | FEMALE | Stage I | T3 | MX | N1b | 3.196274638 | 59.37535607 |
| TCGA-EM-A3FQ | <65 | FEMALE | Stage I | T3 | MX | N1b | 4.310379007 | 12.89020488 |
| TCGA-EM-A3FR | <65 | FEMALE | Stage III | T2 | MX | N1a | 9.700252671 | 27.64058642 |
| TCGA-EM-A3O3 | ≥65 | FEMALE | Stage III | T2 | MX | N1a | 0.194674903 | 26.35460057 |
| TCGA-EM-A3O8 | <65 | FEMALE | Stage I | T1 | MX | N0 | 18.57280692 | 35.98863148 |
| TCGA-EM-A3OA | <65 | FEMALE | Stage II | T2 | MX | N0 | 9.10272943 | 30.85317435 |
| TCGA-EM-A3OB | <65 | FEMALE | Stage I | T2 | MX | N0 | 16.60583001 | 33.92776708 |
| TCGA-EM-A3ST | <65 | FEMALE | Stage III | T3 | MX | N0 | 0.624572342 | 10.40126579 |
| TCGA-EM-A3ST | <65 | FEMALE | Stage III | T3 | MX | N0 | 5.529362536 | 15.70793383 |
| TCGA-EM-A3SU | <65 | FEMALE | Stage I | T2 | MX | N1b | 0.712854231 | 32.96987567 |
| TCGA-EM-A3SU | <65 | FEMALE | Stage I | T2 | MX | N1b | 3.542588839 | 46.48698314 |
| TCGA-EM-A3SX | <65 | FEMALE | Stage I | T3 | MX | N1a | 13.57690308 | 33.65289956 |
| TCGA-EM-A3SZ | <65 | FEMALE | Stage I | T2 | MX | N0 | 7.882613371 | 59.23332928 |
| TCGA-EM-A4FF | <65 | FEMALE | Stage I | T1 | MX | N1 | 7.905087524 | 39.63048932 |
| TCGA-EM-A4FM | <65 | FEMALE | Stage IV | T3 | MX | N1b | 3.293078852 | 36.57590272 |
| TCGA-EM-A4FN | <65 | FEMALE | Stage IV | T1 | MX | N1b | 2.205590736 | 40.12008608 |
| TCGA-EM-A4FO | ≥65 | MALE | Stage I | T1 | MX | N0 | 0.008467583 | 38.82206965 |
| TCGA-EM-A4FQ | <65 | FEMALE | Stage I | T2 | MX | N1 | 6.637402417 | 29.95605428 |
| TCGA-EM-A4FR | <65 | MALE | Stage I | T3 | MX | N1 | 0.001516159 | 19.70865896 |
| TCGA-EM-A4FV | <65 | FEMALE | Stage I | T1 | MX | N0 | 12.50575422 | 40.73496431 |
| TCGA-EM-A4G1 | <65 | FEMALE | Stage I | T1 | MX | N0 | 40.31912722 | 27.76336451 |
| TCGA-ET-A25G | <65 | FEMALE | Stage III | T3 | MX | N0 | 5.180922039 | 57.48384827 |
| TCGA-ET-A25I | <65 | FEMALE | Stage I | T2 | MX | N0 | 3.935128722 | 27.02869932 |
| TCGA-ET-A25K | <65 | FEMALE | Stage I | T2 | MX | N1a | 10.89198314 | 39.76160573 |
| TCGA-ET-A25L | <65 | FEMALE | Stage IV | T3 | MX | N1b | 6.591145487 | 45.26909359 |
| TCGA-ET-A25M | <65 | MALE | Stage I | T2 | MX | N1 | 0.017810001 | 46.46271201 |
| TCGA-ET-A25N | <65 | FEMALE | Stage I | T2 | MX | N1a | 4.161972924 | 38.87921403 |
| TCGA-ET-A25O | <65 | FEMALE | Stage I | T2 | MX | N1a | 17.36566891 | 38.93548543 |
| TCGA-ET-A25P | <65 | FEMALE | Stage I | T1 | MX | N0 | 6.336800498 | 33.06961018 |
| TCGA-ET-A2MX | <65 | MALE | Stage I | T2 | MX | N1a | 0.005630723 | 29.04858101 |
| TCGA-ET-A2MX | <65 | MALE | Stage I | T2 | MX | N1a | 0.011878576 | 21.15798708 |
| TCGA-ET-A2MZ | <65 | MALE | Stage I | T1 | MX | N0 | 0.000625825 | 41.15152145 |
| TCGA-ET-A2N0 | <65 | FEMALE | Stage III | T1 | MX | N1a | 0.040334491 | 11.91545944 |
| TCGA-ET-A2N4 | <65 | FEMALE | Stage III | T3 | MX | N0 | 3.454913559 | 36.48093696 |
| TCGA-ET-A39K | <65 | FEMALE | Stage III | T3 | MX | N1a | 0.459168346 | 21.40645076 |
| TCGA-ET-A39L | <65 | FEMALE | Stage I | T3 | MX | N0 | 6.526510592 | 34.11811062 |
| TCGA-ET-A39O | <65 | MALE | Stage I | T1 | MX | N0 | 0.00582499 | 78.59958416 |
| TCGA-ET-A39P | ≥65 | FEMALE | Stage III | T3 | MX | N0 | 2.149218848 | 48.62151942 |
| TCGA-ET-A39R | <65 | FEMALE | Stage I | T1 | MX | N0 | 8.421229294 | 69.51904786 |
| TCGA-ET-A39S | <65 | FEMALE | Stage I | T1 | MX | N0 | 2.498986914 | 42.71563686 |
| TCGA-ET-A39T | <65 | FEMALE | Stage III | T3 | MX | N0 | 8.297376948 | 44.19903423 |
| TCGA-ET-A3BO | <65 | FEMALE | Stage I | T3 | MX | N1a | 10.48299995 | 56.74896625 |
| TCGA-ET-A3BP | <65 | FEMALE | Stage I | T2 | MX | N1a | 5.273005222 | 57.66010652 |
| TCGA-ET-A3BQ | <65 | FEMALE | Stage I | T2 | MX | N0 | 3.12596474 | 44.87175989 |
| TCGA-ET-A3BS | <65 | MALE | Stage I | T3 | MX | N1a | 0.003674508 | 39.88133026 |
| TCGA-ET-A3BT | <65 | FEMALE | Stage IV | T2 | MX | N1b | 2.512818671 | 51.33267825 |
| TCGA-ET-A3BU | <65 | MALE | Stage I | T1 | MX | N1a | 0.442664376 | 52.00618808 |
| TCGA-ET-A3BV | <65 | FEMALE | Stage III | T2 | MX | N1a | 5.392483844 | 46.54988981 |
| TCGA-ET-A3BX | <65 | MALE | Stage I | T3 | MX | N1a | 0 | 45.10951032 |
| TCGA-ET-A3DQ | <65 | FEMALE | Stage I | T1 | MX | N0 | 4.194690528 | 52.7610411 |
| TCGA-ET-A3DR | <65 | FEMALE | Stage I | T1 | MX | N1a | 9.117980257 | 33.63263295 |
| TCGA-ET-A3DS | <65 | FEMALE | Stage I | T1 | MX | N0 | 8.890618336 | 33.3738073 |
| TCGA-ET-A3DU | <65 | FEMALE | Stage I | T1 | MX | N1a | 1.881879984 | 44.12818624 |
| TCGA-ET-A3DV | ≥65 | FEMALE | Stage II | T3 | MX | N0 | 0.444568787 | 20.40256395 |
| TCGA-ET-A3DW | <65 | MALE | Stage III | T2 | MX | N1 | 0.002942488 | 47.83333884 |
| TCGA-ET-A3DW | <65 | MALE | Stage III | T2 | MX | N1 | 0.002387333 | 29.25490026 |
| TCGA-ET-A40P | <65 | FEMALE | Stage I | T2 | MX | N0 | 1.396345355 | 28.87595175 |
| TCGA-ET-A40Q | <65 | MALE | Stage I | T3 | MX | N1b | 0.011373747 | 78.97093275 |
| TCGA-ET-A40R | <65 | FEMALE | Stage I | T2 | MX | N1a | 1.785730269 | 32.79485513 |
| TCGA-ET-A40S | <65 | MALE | Stage II | T2 | MX | N0 | 0.004112737 | 74.79393558 |
| TCGA-ET-A40T | <65 | FEMALE | Stage I | T2 | MX | N1a | 13.95698217 | 54.66367903 |
| TCGA-ET-A4KN | <65 | FEMALE | Stage III | T3 | MX | N0 | 0.064381666 | 7.783322462 |
| TCGA-FE-A22Z | <65 | FEMALE | Stage IV | T4 | MX | N1 | 7.961856095 | 50.45609614 |
| TCGA-FE-A230 | <65 | FEMALE | Stage I | T3 | MX | N1a | 8.928556978 | 80.49473632 |
| TCGA-FE-A231 | ≥65 | MALE | Stage IV | T3 | MX | N1b | 0.009527413 | 86.12063591 |
| TCGA-FE-A234 | <65 | FEMALE | Stage I | T2 | MX | N1 | 13.10346068 | 75.44208322 |
| TCGA-FE-A235 | <65 | FEMALE | Stage I | T2 | MX | N1 | 0.517878825 | 42.54472357 |
| TCGA-FE-A236 | <65 | MALE | Stage I | T2 | MX | N1 | 0.018966054 | 54.65805797 |
| TCGA-FE-A237 | <65 | FEMALE | Stage I | T3 | MX | N1 | 9.173947807 | 46.12637342 |
| TCGA-FE-A238 | <65 | FEMALE | Stage I | T3 | MX | N0 | 7.74641503 | 53.09250958 |
| TCGA-FE-A239 | ≥65 | MALE | Stage III | T3 | MX | N0 | 0.018124545 | 43.8930474 |
| TCGA-FE-A23A | <65 | FEMALE | Stage I | T2 | MX | N0 | 7.211622651 | 30.65748775 |
| TCGA-FE-A3PA | <65 | MALE | Stage II | T4 | M1 | N0 | 0.002132081 | 31.21983891 |
| TCGA-FE-A3PB | <65 | FEMALE | Stage I | T3 | MX | N1a | 8.864097565 | 43.68772142 |
| TCGA-FE-A3PC | <65 | FEMALE | Stage I | T3 | MX | N1 | 6.017742118 | 19.41569385 |
| TCGA-FE-A3PD | <65 | FEMALE | Stage I | T2 | MX | N0 | 8.163993621 | 62.34401792 |
| TCGA-FK-A3S3 | <65 | FEMALE | Stage I | T3 | MX | N1b | 1.358656937 | 32.95906005 |
| TCGA-FK-A3SB | <65 | FEMALE | Stage I | T3 | MX | N1a | 8.009438196 | 34.80044593 |
| TCGA-FK-A3SD | <65 | FEMALE | Stage I | T1 | M0 | N0 | 0.592653264 | 18.29805249 |
| TCGA-FK-A3SE | <65 | FEMALE | Stage I | T2 | MX | N1a | 4.41092855 | 65.80543666 |
| TCGA-FK-A3SG | <65 | FEMALE | Stage I | T2 | MX | N1b | 2.238137331 | 46.5805943 |
| TCGA-FK-A3SH | <65 | FEMALE | Stage III | T2 | MX | N1a | 2.115170607 | 29.03976322 |
| TCGA-FK-A4UB | <65 | MALE | Stage III | T3 | MX | N1a | 0.006431045 | 24.4298059 |
| TCGA-FY-A3I4 | <65 | FEMALE | Stage I | T2 | M0 | N0 | 6.765144562 | 24.15839283 |
| TCGA-FY-A3I5 | <65 | FEMALE | Stage III | T3 | MX | N0 | 12.87288586 | 42.63794126 |
| TCGA-FY-A3NM | <65 | FEMALE | Stage III | T3 | MX | N0 | 10.05478447 | 40.57835107 |
| TCGA-FY-A3NN | <65 | FEMALE | Stage III | T1 | MX | N1a | 5.044584794 | 57.40160035 |
| TCGA-FY-A3NP | ≥65 | MALE | Stage I | T1 | M0 | N0 | 0 | 26.84181758 |
| TCGA-FY-A3ON | <65 | MALE | Stage I | T2 | MX | N1 | 0.001508287 | 37.88784553 |
| TCGA-FY-A3R6 | <65 | FEMALE | Stage IV | T2 | MX | N1b | 3.150545932 | 58.13259518 |
| TCGA-FY-A3R7 | <65 | FEMALE | Stage III | T3 | MX | N1a | 5.732445168 | 45.07478398 |
| TCGA-FY-A3R9 | ≥65 | FEMALE | Stage I | T1 | MX | N0 | 14.65012612 | 32.36413573 |
| TCGA-FY-A3TY | <65 | FEMALE | Stage III | T3 | MX | N0 | 33.51826168 | 26.16940438 |
| TCGA-FY-A3TY | <65 | FEMALE | Stage III | T3 | MX | N0 | 6.861875138 | 50.76445055 |
| TCGA-FY-A3W9 | ≥65 | FEMALE | Stage II | T2 | MX | N0 | 29.46156355 | 24.85303591 |
| TCGA-FY-A3YR | <65 | FEMALE | Stage III | T3 | MX | N1a | 15.13014822 | 54.23037911 |
| TCGA-FY-A40K | <65 | FEMALE | Stage I | T1 | MX | N0 | 25.82891676 | 38.82359117 |
| TCGA-FY-A40L | <65 | FEMALE | Stage IV | T4 | MX | N1b | 12.53160987 | 47.52798068 |
| TCGA-FY-A40M | <65 | FEMALE | Stage I | T1 | MX | N0 | 18.74658948 | 26.70819754 |
| TCGA-FY-A40N | <65 | FEMALE | Stage I | T1 | MX | N0 | 29.54288467 | 28.13533251 |
| TCGA-FY-A4B3 | <65 | MALE | Stage III | T3 | MX | N1a | 0.001486478 | 51.91480643 |
| TCGA-FY-A4B4 | <65 | FEMALE | Stage III | T1 | MX | N1a | 9.997591244 | 84.39331619 |
| TCGA-GE-A2C6 | <65 | FEMALE | Stage I | T2 | MX | N1b | 15.2513479 | 41.70568805 |
| TCGA-GE-A2C6 | <65 | FEMALE | Stage I | T2 | MX | N1b | 18.28771034 | 33.83913676 |
| TCGA-H2-A26U | <65 | FEMALE | Stage III | T3 | MX | N0 | 9.524642022 | 64.38312908 |
| TCGA-H2-A2K9 | <65 | MALE | Stage I | T2 | MX | N1b | 0.014391276 | 32.59840156 |
| TCGA-H2-A2K9 | <65 | MALE | Stage I | T2 | MX | N1b | 0.010092593 | 38.58666036 |
| TCGA-H2-A3RH | <65 | FEMALE | Stage I | T1 | M0 | N0 | 24.56152682 | 37.67008045 |
| TCGA-H2-A3RI | <65 | FEMALE | Stage I | T3 | MX | N0 | 19.02131598 | 39.42433515 |
| TCGA-H2-A421 | <65 | FEMALE | Stage I | T1 | M0 | N1a | 11.65207333 | 38.33019145 |
| TCGA-H2-A422 | <65 | FEMALE | Stage I | T2 | MX | N0 | 24.48801581 | 44.67615538 |
| TCGA-IM-A3EB | <65 | FEMALE | Stage I | T3 | MX | N1a | 1.875313242 | 112.8943034 |
| TCGA-IM-A3ED | <65 | FEMALE | Stage I | T1 | MX | N0 | 7.294888896 | 57.37595805 |
| TCGA-IM-A3U2 | ≥65 | FEMALE | Stage IV | T3 | MX | N1b | 1.266438487 | 35.90222285 |
| TCGA-IM-A3U3 | <65 | FEMALE | Stage I | T1 | MX | N0 | 13.08197767 | 41.86962982 |
| TCGA-IM-A41Y | <65 | FEMALE | Stage I | T3 | MX | N1a | 9.005958611 | 40.47293724 |
| TCGA-IM-A41Z | <65 | FEMALE | Stage I | T1 | MX | N0 | 3.297466715 | 29.22476865 |
| TCGA-IM-A420 | <65 | FEMALE | Stage I | T2 | MX | N1a | 14.30626826 | 44.4998287 |
| TCGA-IM-A4EB | <65 | MALE | Stage I | T3 | MX | N1b | 0.032628504 | 29.63741373 |
| TCGA-J8-A3NZ | <65 | FEMALE | Stage III | T3 | M0 | N1a | 11.867939 | 66.34212348 |
| TCGA-J8-A3O0 | <65 | MALE | Stage I | T2 | M0 | N0 | 0.002956129 | 37.53187254 |
| TCGA-J8-A3O1 | <65 | FEMALE | Stage I | T3 | M0 | N1b | 11.19925276 | 40.88474326 |
| TCGA-J8-A3O2 | <65 | MALE | Stage I | T3 | M0 | N1b | 0.017954829 | 30.45353183 |
| TCGA-J8-A3O2 | <65 | MALE | Stage I | T3 | M0 | N1b | 0.011223155 | 42.71593924 |
| TCGA-J8-A3YD | <65 | FEMALE | Stage III | T1 | M0 | N1a | 16.19674214 | 34.44290603 |
| TCGA-J8-A3YE | <65 | FEMALE | Stage I | T3 | M0 | N1 | 20.4511323 | 67.89481791 |
| TCGA-J8-A3YF | ≥65 | MALE | Stage III | T1 | M0 | N1 | 0.012215599 | 37.15432713 |
| TCGA-J8-A3YG | <65 | FEMALE | Stage III | T2 | M0 | N1a | 18.60765184 | 52.27171862 |
| TCGA-J8-A3YH | <65 | MALE | Stage I | T3 | M0 | N1b | 0.008982308 | 35.88810911 |
| TCGA-J8-A3YH | <65 | MALE | Stage I | T3 | M0 | N1b | 0 | 37.04016769 |
| TCGA-J8-A42S | <65 | MALE | Stage III | T1 | MX | N1a | 0.055241926 | 53.91764401 |
| TCGA-J8-A4HW | <65 | FEMALE | Stage III | T2 | M0 | N1 | 9.596200854 | 63.51475757 |
| TCGA-J8-A4HW | <65 | FEMALE | Stage III | T2 | M0 | N1 | 19.10858533 | 45.40233802 |
| TCGA-J8-A4HY | ≥65 | FEMALE | Stage IV | T4 | MX | N1b | 3.497185924 | 30.43113544 |
| TCGA-KS-A41F | <65 | FEMALE | Stage I | T2 | M0 | N1 | 1.328979775 | 7.723832859 |
| TCGA-KS-A41I | <65 | FEMALE | Stage I | T1 | M0 | N0 | 19.20942316 | 37.86050987 |
| TCGA-KS-A41I | <65 | FEMALE | Stage I | T1 | M0 | N0 | 3.700771365 | 9.823663271 |
| TCGA-KS-A41J | <65 | FEMALE | Stage I | T2 | M0 | N1 | 24.85073063 | 37.80307732 |
| TCGA-KS-A41J | <65 | FEMALE | Stage I | T2 | M0 | N1 | 0.428260174 | 24.34534172 |
| TCGA-KS-A41L | <65 | FEMALE | Stage I | T2 | M0 | N0 | 31.58958668 | 30.98655994 |
| TCGA-KS-A41L | <65 | FEMALE | Stage I | T2 | M0 | N0 | 6.316564481 | 15.05424483 |
| TCGA-KS-A4I1 | <65 | FEMALE | Stage I | T2 | M0 | N0 | 2.761939634 | 29.60188717 |
| TCGA-KS-A4I3 | <65 | MALE | Stage I | T3 | M0 | N1 | 0.008172743 | 30.76934143 |
| TCGA-KS-A4I5 | <65 | FEMALE | Stage IV | T1 | M0 | N1b | 5.427649578 | 31.4349866 |
| TCGA-KS-A4I7 | <65 | FEMALE | Stage I | T1 | M0 | N0 | 4.304312025 | 57.6477138 |
| TCGA-KS-A4I9 | <65 | FEMALE | Stage I | T1 | M0 | N0 | 12.2601762 | 52.27709961 |
| TCGA-KS-A4IB | <65 | FEMALE | Stage I | T2 | M0 | N1 | 15.20647588 | 31.43245342 |
| TCGA-KS-A4IC | <65 | FEMALE | Stage III | T1 | M0 | N1a | 4.013377772 | 43.85572077 |
| TCGA-KS-A4ID | <65 | FEMALE | Stage I | T1 | M0 | N0 | 8.218877444 | 36.26157278 |
| TCGA-L6-A4EP | <65 | FEMALE | Stage I | T2 | M0 | N0 | 1.526948764 | 28.54118833 |
| TCGA-L6-A4EQ | <65 | MALE | Stage III | T2 | M0 | N1a | 0.010984484 | 45.14180251 |
| TCGA-L6-A4EU | <65 | FEMALE | Stage III | T3 | M0 | N1a | 1.209317637 | 53.91617821 |
| TCGA-MK-A4N6 | <65 | MALE | Stage I | T3 | M0 | N1b | 0.016320856 | 124.6240538 |
| TCGA-MK-A4N7 | <65 | FEMALE | Stage I | T2 | M0 | N0 | 15.21200535 | 61.87501311 |
| TCGA-MK-A4N9 | <65 | FEMALE | Stage I | T3 | M0 | N1 | 23.12110982 | 47.3417863 |
| TCGA-QD-A8IV | <65 | FEMALE | Stage IV | T3 | MX | N1b | 12.57279422 | 28.49946412 |
