# Supplementary material for: Artemisinin suppresses aerobic glycolysis in thyroid cancer cells by downregulating HIF-1a, which is increased by the XIST/miR-93/HIF-1a pathway
Source: PLoS One. 2023 Apr 10;18(4):e0284242. doi: 10.1371/journal.pone.0284242 (PMC10085032; doi:10.1371/journal.pone.0284242)
Supplement: S2 Table — (DOCX) [file pone.0284242.s002.docx]

**S2 Table. Patient information of the Hebei Medical University Fourth Hospital.**

| ID | sex | AGE | TNM | T | N | XISTC | XISTN | XISTV | miR93C | miR93N | miR93V |
| --- | --- | --- | --- | --- | --- | --- | --- | --- | --- | --- | --- |
| 539562 | 1 | 67 | 3 | 4 | 1 | 1 | 1 | 1 | 1 | 1 | 1 |
| 542475 | 1 | 70 | 3 | 2 | 1 | 3.554385103 | 0.897870945 | 3.958681504 | 4.469024643 | 0.452785606 | 9.870067835 |
| 546069 | 1 | 72 | 1 | 3 | 0 | 4.967327955 | 1.687432742 | 2.943719078 | 1.039082353 | 1.092853487 | 0.95079749 |
| 552126 | 2 | 71 | 1 | 2 | 0 | 2.865327411 | 4.424241076 | 0.647642694 | 2.522597445 | 3.74223459 | 0.674088538 |
| 552297 | 1 | 61 | 3 | 3 | 1 | 9.018765693 | 2.609584286 | 3.456016248 | 1.87515519 | 9.666542747 | 0.193984058 |
| 558665 | 2 | 65 | 3 | 3 | 2 | 8.729324389 | 2.785371963 | 3.133988748 | 2.607794163 | 2.43479448 | 1.071053095 |
| 559108 | 1 | 66 | 3 | 3 | 1 | 6.242829221 | 3.785909698 | 1.648964112 | 2.848554483 | 0.600901322 | 4.740469658 |
| 559432 | 1 | 65 | 2 | 3 | 0 | 6.242829221 | 3.785909698 | 1.648964112 | 8.378314266 | 0.805468458 | 10.40179064 |
| 559519 | 1 | 78 | 3 | 3 | 1 | 7.042853143 | 3.834052255 | 1.836921532 | 2.888078588 | 1.359145586 | 2.12492217 |
| 559637 | 2 | 71 | 3 | 3 | 1 | 16.17495732 | 4.649385337 | 3.478945311 | 8.068832828 | 1.033830736 | 7.804791002 |
| 560672 | 2 | 68 | 3 | 4 | 1 | 2.609801355 | 7.059959953 | 0.369662345 | 2.355968092 | 1.224251531 | 1.924415067 |
| 560702 | 2 | 66 | 3 | 3 | 1 | 14.18810493 | 10.28321869 | 1.379733851 | 3.513989791 | 0.76774547 | 4.577024454 |
| 560708 | 1 | 72 | 3 | 3 | 1 | 14.18810493 | 10.28321869 | 1.379733851 | 4.447331667 | 1.111879158 | 3.999833648 |
| 560735 | 1 | 87 | 3 | 4 | 2 | 19.3523921 | 5.866581178 | 3.298751268 | 2.571178909 | 1.964551516 | 1.308786707 |
| 560864 | 1 | 60 | 3 | 3 | 2 | 43.7830332 | 9.529034203 | 4.594697875 | 2.571178909 | 2.069955854 | 1.242141905 |
| 561379 | 1 | 67 | 3 | 3 | 2 | 16.18673381 | 7.044464299 | 2.297794853 | 15.68539548 | 4.691339797 | 3.34347887 |
| 561421 | 1 | 59 | 2 | 3 | 0 | 23.37784433 | 0.872525984 | 26.793293 | 3.026897874 | 239.5030138 | 0.012638245 |
| 561435 | 1 | 64 | 3 | 3 | 1 | 28.39746021 | 0.78626089 | 36.11709619 | 6.653622427 | 239.5030138 | 0.027780955 |
| 561483 | 1 | 75 | 3 | 4 | 0 | 28.39746021 | 0.78626089 | 36.11709619 | 4.431238567 | 239.5030138 | 0.018501807 |
| 561693 | 2 | 71 | 3 | 3 | 1 | 41.14342524 | 0.973545289 | 42.26143939 | 2.256927382 | 9.571201224 | 0.235803984 |
| 561733 | 1 | 63 | 3 | 3 | 1 | 4.016196896 | 4.53489768 | 0.885620179 | 4.790313249 | 3.74223459 | 1.2800676 |
| 562569 | 2 | 67 | 3 | 3 | 1 | 4.135867594 | 1.666267468 | 2.48211507 | 4.790313249 | 1.789429865 | 2.677005309 |
| 563279 | 1 | 76 | 2 | 3 | 0 | 2.812844891 | 4.293160176 | 0.65519216 | 7.062407173 | 3.74223459 | 1.887216582 |
| 563460 | 1 | 69 | 2 | 3 | 0 | 4.763493433 | 5.483473286 | 0.868700035 | 6.376744159 | 3.74223459 | 1.703993698 |
| 563515 | 1 | 66 | 3 | 3 | 2 | 7.335066269 | 12.93967969 | 0.566866139 | 5.879445098 | 12.86785786 | 0.456909391 |
| 563846 | 1 | 61 | 3 | 3 | 2 | 7.335066269 | 12.93967969 | 0.566866139 | 6.517234924 | 3.143343639 | 2.073344716 |
| 564602 | 1 | 61 | 3 | 3 | 1 | 8.039857884 | 9.516822742 | 0.844804837 | 9.223088875 | 3.74223459 | 2.464593989 |
| 564802 | 1 | 68 | 1 | 2 | 0 | 13.01686388 | 10.07878621 | 1.291511062 | 1.152886109 | 3.74223459 | 0.308074249 |
| 565924 | 2 | 60 | 2 | 3 | 0 | 7.975085578 | 12.28392752 | 0.649229293 | 1.152886109 | 3.74223459 | 0.308074249 |
| 566530 | 1 | 57 | 3 | 3 | 2 | 14.35697598 | 13.05707644 | 1.099555175 | 1.028619921 | 3.74223459 | 0.274867835 |
| 567275 | 1 | 66 | 3 | 3 | 1 | 7.975085578 | 12.28392752 | 0.649229293 | 1.02080691 | 3.74223459 | 0.272780042 |
| 567865 | 1 | 64 | 3 | 3 | 2 | 14.35697598 | 13.05707644 | 1.099555175 | 0.685343896 | 3.74223459 | 0.183137609 |
| 567974 | 1 | 61 | 3 | 4 | 2 | 14.35697598 | 13.05707644 | 1.099555175 | 2.049582411 | 3.74223459 | 0.547689452 |
| 568021 | 1 | 73 | 3 | 3 | 2 | 18.26360933 | 50.60121075 | 0.36093226 | 2.109761621 | 7.495267668 | 0.281479157 |
| 568116 | 1 | 61 | 1 | 1 | 0 | 6.138856729 | 12.51555185 | 0.490498286 | 1.56311075 | 2.919659243 | 0.535374378 |
| 568136 | 1 | 64 | 3 | 3 | 1 | 10.51917751 | 53.61073465 | 0.196214015 | 1.430212978 | 3.270996011 | 0.43724082 |
| 568657 | 2 | 63 | 1 | 1 | 0 | 9.59584597 | 8.278273996 | 1.159160228 | 1.430212978 | 3.74223459 | 0.38218154 |
| 568854 | 1 | 67 | 3 | 3 | 2 | 9.59584597 | 49.87586648 | 0.192394572 | 2.544566685 | 21.84225647 | 0.116497427 |
| 568902 | 2 | 72 | 3 | 3 | 3 | 14.90474719 | 13.26331961 | 1.12375692 | 1.073944918 | 3.74223459 | 0.286979582 |
| 569002 | 1 | 70 | 3 | 3 | 1 | 0.624710637 | 0.274352 | 2.277040576 | 0.973484558 | 0.809116866 | 1.203144564 |
| 569141 | 1 | 73 | 3 | 3 | 1 | 0.750555156 | 0.274352 | 2.735737866 | 0.806736815 | 0.809116866 | 0.997058459 |
| 569683 | 1 | 59 | 3 | 4 | 1 | 0.750555156 | 0.490692117 | 1.529584703 | 0.509036143 | 1.57649435 | 0.322891194 |
| 569844 | 1 | 59 | 2 | 2 | 0 | 1.143899257 | 0.376322121 | 3.039681152 | 1.061783774 | 0.499279646 | 2.126631402 |
| 570686 | 1 | 71 | 3 | 3 | 2 | 1.109600236 | 0.056979233 | 19.47376591 | 1.369424758 | 0.462594494 | 2.960313568 |
| 570906 | 1 | 68 | 2 | 3 | 0 | 1.127345712 | 0.056979233 | 19.78520351 | 1.146733382 | 2.970941093 | 0.385983211 |
| 571599 | 1 | 67 | 3 | 4 | 0 | 1.223182779 | 0.034638254 | 35.31306157 | 1.046723214 | 0.562849064 | 1.859687226 |
| 571767 | 1 | 61 | 1 | 1 | 0 | 1.223182779 | 0.055918908 | 21.87422503 | 1.046723214 | 11.0151711 | 0.095025597 |
| 572211 | 1 | 71 | 2 | 2 | 0 | 1.200270852 | 0.055918908 | 21.46449016 | 1.728151861 | 3.315025574 | 0.521308757 |
| 573373 | 1 | 76 | 3 | 3 | 1 | 1.200270852 | 0.030821054 | 38.94321265 | 1.250764346 | 1.014325014 | 1.233100169 |
| 574464 | 1 | 65 | 3 | 3 | 1 | 1.425017859 | 0.030821054 | 46.23520884 | 2.482803354 | 2.929227002 | 0.847596773 |
| 576603 | 1 | 68 | 3 | 4 | 0 | 0.916476631 | 0.027419385 | 33.42440561 | 1.577401588 | 1.208167095 | 1.305615419 |
| 577758 | 1 | 65 | 3 | 3 | 2 | 0.916476631 | 0.038066085 | 24.07593631 | 1.216174293 | 0.499279646 | 2.435857945 |
| 578083 | 2 | 58 | 2 | 3 | 0 | 0.618163339 | 0.038066085 | 16.23921512 | 1.606075818 | 0.499279646 | 3.216786084 |
| 578526 | 1 | 75 | 2 | 3 | 0 | 0.618163339 | 0.028390459 | 21.77362937 | 5.888744176 | 0.620077308 | 9.496790316 |
| 579479 | 1 | 63 | 3 | 3 | 1 | 0.603920269 | 0.028390459 | 21.27194431 | 4.011606226 | 0.620077308 | 6.469525934 |
| 580082 | 2 | 67 | 1 | 1 | 0 | 0.63916486 | 0.077753423 | 8.220407998 | 6.865286844 | 0.902200506 | 7.609491238 |
