# Supplementary figures and images for: Artemisinin suppresses aerobic glycolysis in thyroid cancer cells by downregulating HIF-1a, which is increased by the XIST/miR-93/HIF-1a pathway
Source: PLoS One. 2023 Apr 10;18(4):e0284242. doi: 10.1371/journal.pone.0284242 (PMC10085032; doi:10.1371/journal.pone.0284242)

HIF-1A

GAPDH

Control

Artemisinin

HIF-1A

GAPDH

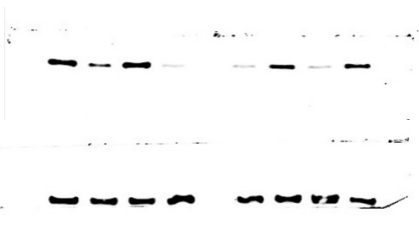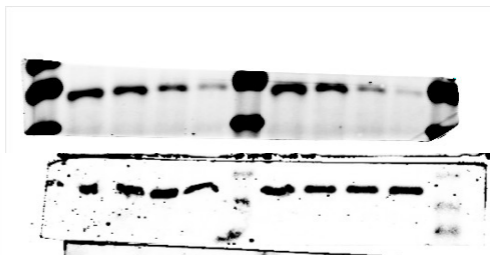

Supplement: S1 Raw images — (PDF) [file pone.0284242.s003.pdf]
